# Discrete Fourier Transform Windowing Techniques for Cerebral Physiological Research in Neural Injury: A Practical Demonstration

**DOI:** 10.1089/neur.2022.0079

**Published:** 2023-06-22

**Authors:** Logan Froese, Amanjyot Singh Sainbhi, Alwyn Gomez, Izzy Marquez, Fiorella Amenta, Carleen Batson, Kevin Y. Stein, Frederick A. Zeiler

**Affiliations:** ^1^ Biomedical Engineering, Rady Faculty of Health Sciences, University of Manitoba, Winnipeg, Manitoba, Canada.; ^2^ Section of Neurosurgery, Department of Surgery, Rady Faculty of Health Sciences, University of Manitoba, Winnipeg, Manitoba, Canada.; ^3^ Department of Human Anatomy and Cell Science, Rady Faculty of Health Sciences, University of Manitoba, Winnipeg, Manitoba, Canada.; ^4^ Undergraduate Engineering Program, Department of Biosystems Engineering, Price Faculty of Engineering, Rady Faculty of Health Sciences, University of Manitoba, Winnipeg, Manitoba, Canada.; ^5^ Department of Clinical Neuroscience, Karolinska Institutet, Stockholm, Sweden.; ^6^ Division of Anaesthesia, Department of Medicine, Addenbrooke's Hospital, University of Cambridge, Cambridge, United Kingdom.

**Keywords:** blood flow, Fourier transform, models of injury, pulse amplitude, traumatic brain injury, waveform

## Abstract

To optimally assess oscillatory phenomena within physiological variables, spectral domain transforms are used. A discrete Fourier transform (DFT) is one of the most common methods used to attain this spectral change. In traumatic brain injury (TBI), a DFT is used to derive more complicated methods of physiological assessment, particularly that of cerebrovascular reactivity (CVR). However, a practical application of a DFT will introduce various errors that need to be considered. This study will evaluate the pulse amplitude DFT derivation of intracranial pressure (AMP) to highlight how slight differences in DFT methodologies can impact calculations. Utilizing a high-frequency prospectively maintained data set of TBI patients with recorded arterial and intracranial blood pressure, various cerebral physiological aspects of interest were assessed using the DFT windowing methods of rectangular, Hanning, and Chebyshev. These included AMP, CVR indices (including the pressure reactivity and pulse amplitude index), and the optimal cerebral perfusion pressure (with all methods of CVR). The results of the different DFT-derived windowing methods were compared using the Wilcoxon signed-ranked test and histogram plots between individual patients and over the whole 100-patient cohort. The results for this analysis demonstrate that, overall and for grand average values, there were limited differences between the different DFT windowing techniques. However, there were individual patient outliers to whom the different methods resulted in noticeably different overall values. From this information, for derived indices utilizing a DFT in the assessment of AMP, there are limited differences within the resulting calculations for larger aggregates of data. However, when the amplitude of spectrally resolved response is important and needs to be robust in smaller moments in time, it is recommended to use a window that has amplitude accuracy (such as Chebyshev or flat-top).

## Introduction

Many modern approaches to healthcare have long utilized complex computational techniques to better conceptualize pathophysiological relationships and response in patient care. In order to assess oscillatory physiology, it is often an advantage to convert time-domain signals into the spectral domain; such methods include heart-rate variability and cerebrovascular reactivity (CVR).^1,2^ In these spectral domain resolved analyses, the ability to better conceptualize physiological relationships can occur.

Of the mathematical methods used to convert time-domain signals into a spectral domain, often leveraged is a Fourier transform (FT), or a more practical method of a discrete FT (DFT). CVR has recently been derived with a DFT using pulse amplitude-derived intracranial pressure (ICP; AMP) and arterial blood pressure (ABP), called the pulse amplitude index (PAx).^3–7^ Within traumatic brain injury (TBI) critical care, there has been recent work that has documented that DFT-derived measures of CVR perform differently than time-domain measures.^2,8,9^ However, limited work has been given to the practical implications of different DFT methodologies and the resulting physiological measures.

Thus, to assess the application of a DFT in CVR, different windowing methods of DFT-derived PAx will be compared in a TBI population. Through this assessment, the goal is to foster a base understanding of a DFT and a clear overview of the currently utilized techniques, pitfalls, and other associated concepts. Finally, by assessing different DFT windowing methods, the nuanced response with each method can be observed and further highlight which method of DFT windowing is the best for a given application.

## Methods

### Study design

From a prospectively maintained TBI database at the Winnipeg Acute TBI Laboratories, at the University of Manitoba, those patients with archived high-frequency digital physiology (ICP and arterial blood pressure [ABP]) were used (from 2018 to 2022). All patients included in this database are age ≥17 years, who have suffered moderate-to-severe TBI, requiring admission to the surgical intensive care unit for invasive ICP monitoring. Patients received treatment according to the Brain Trauma Foundation guidelines.^10^

### Ethics

Local research ethics board approval at the University of Manitoba is in place for all aspects of this database (H2017:181, H2017:188 and H2020:118).

### Patient data collection

This methodology is taken from our previous studies with all patients having high-frequency digital signals recorded throughout their intensive care unit stay.^11–14^ ABP was obtained through radial or femoral arterial lines connected to pressure transducers (Baxter Healthcare Corp. CardioVascular Group, Irvine, CA, or similar devices). ICP was acquired by an intraparenchymal strain gauge probe (Codman ICP MicroSensor; Codman & Shurtlef Inc., Raynham, MA). These signals were captured simultaneously and digitized by an A/D converter (DT9804; Data Translation, Marlboro, MA), sampled at a frequency of ≥100 Hz, using Intensive Care Monitoring (ICM+) software (Cambridge Enterprise Ltd, Cambridge, UK). All signal artifacts were removed using manual methods before further processing and analysis.

### Clinical primer of Fourier transform

A more detailed description about a FT and its practical pitfalls, including demonstrations of the overarching concepts, can be found in Supplementary Appendices SA and SB. As a summary, an FT allows for any continuous signal to be recreated by a series sum of sine waves.^15^ In order to apply an FT to practical applications, a DFT is used (a computational FT application). Using a DFT to convert a time-domain system to a spectral domain, aspects of physiological response (particularly oscillatory responses) become more apparent. Thus, whenever oscillatory phenomena are being evaluated, the use of a DFT or other domain-altering transforms are commonly used. However, because a DFT is altering a real-time domain system to the spectral domain, various factors should be assessed—things like the window of time being assessed, the frequency of the data being sampled, and the windowing method applied.

Choosing an optimal sampling rate can help reduce spectral leakage by selecting a rate whose frequency component is a factor of the sampling rate. Moreover, at a minimum, the sampling rate should be chosen to be ≥4–10 times the desired frequency (note this assumes that the signal is a perfect sine wave).^16^

The window size can play a role in the accuracy of the spectral domain. Like the sampling rate, if the desired frequency component is known, choosing a window size that is a factor of this frequency component is best. However, most applications of a DFT require the window length to be ≥0.5 the length of the slowest component assessed.

Finally, the windowing method used to perform a DFT will influence the two components of a spectral domain representation (i.e., its frequency and amplitude of these frequency components). Various errors, including spectral leakage, scalloping, magnitude modification, and bandwidth noise, are all influenced by the window chosen (for more details on these errors, see Supplementary Appendices SA and SB).

Within this article, only the optimal windowing methods will be discussed, given that sampling rate and windowing size are often fixed, because the collection frequency and oscillatory physiology are bound to the data itself. To this end, we will highlight the major differences between the DFT windows of rectangular, Hanning, and Chebyshev. The rectangular window is the most accurate at differentiating frequency, Chebyshev is one of the best at differentiating amplitude (also more commonly used than flat-top), and Hanning is one of the more commonly utilized windows that has a balance between the two other windows (see Table 1 for more windowing types).

**Table 1.** Windows

Window	Frequency resolution	Amplitude accuracy	Spectral leakage	Best physiological aspect uses
Rectangular/boxcar/uniform/none	Best	Poor	Poor	Optimal when determining the key frequency components of a value - Heart rate or oscillatory rate of fundamental physiology - Broadband random, closely spaced sine waves, signals with multiple tones and equal amplitudes
Hanning	Good	Good	Fair	This is one of the more common windows used, it has a balance between all general categories -Basic single/multiple sine waves -Narrowband random -Vibration data
Hamming	Good	Fair	Fair	Better at frequency distinction than Hanning for closely spaced sine waves with different amplitudes and better at amplitude resolution than rectangular -Physiological responses in multiple sine waves with close frequencies and where amplitude resolution is important
Kaiser-Bessel/Kaiser	Good	Fair	Fair	Excellent for distinguishing two tones with close frequencies but different amplitudes
Bartlett	Good	Fair	Fair	Used to taper a signal, which means to reduce the overall energy of all components without segmenting
Blackman	Fair	Good	Best	Great for ensuring amplitude accuracy of closely spaced frequency components - Physiological variables that are closely spaced and the distinguishing characteristic is amplitude
Dolph-Chebyshev/Chebyshev	Poor	Good	Good	Is effective at determining sinewave amplitude - Single wave (in filtered range) pulse amplitude indices
Exponential/Poison (note this can vary significantly)	Good	Poor	Poor	Because this window can be more significantly modified than the other methods, it can be used to best distinguish a response signal (a pulse or momentary change)
Flat-top	Poor	Best	Good	This is the best filter to determine amplitude accuracy - A single sine wave amplitude accuracy

### Signal processing

Mean arterial blood pressure (MAP) and ICP were calculated as the mean value over a 10-sec window, from the ABP and ICP waves. Cerebral perfusion pressure (CPP) was derived as MAP – ICP. The pulse amplitude of an ICP wave (AMP) is derived over a 10-sec window of ICP using different DFT windows (see the subsection above for more details on the DFT analyses).^3–7^ Pressure reactivity index (PRx) is the most common method of CVR and is determined through a Pearson's correlation between ICP and ABP.^17–19^ PAx methodology is taken from the methods outlined by Aries and colleagues and Zeiler and colleagues by correlating AMP and MAP.^3–7^ PRx and PAx are both surrogate measures for the cerebral autoregulatory state in TBI patients, resulting in values from −1 to 1, with those above ∼0.25 indicating impaired states^2,8,9^; however, PAx uses a DFT to find AMP and has shown some slight overall differences in response.^2,8,9^

Finally, optimal cerebral perfusion pressure (CPPopt) was found using the OPT Flex methodologies, which links the lowest PRx/PAx values (most intact CVR) to its associated CPP.^17,20–22^ For this method, PRx/PAx values were divided and averaged into CPP bins spanning 5 mm Hg. Then an automatic curve fitting method was applied to the binned CPP data to determine the CPP value with the lowest associated PRx/PAx value.

### Statistical analysis

**FIG. 1.**
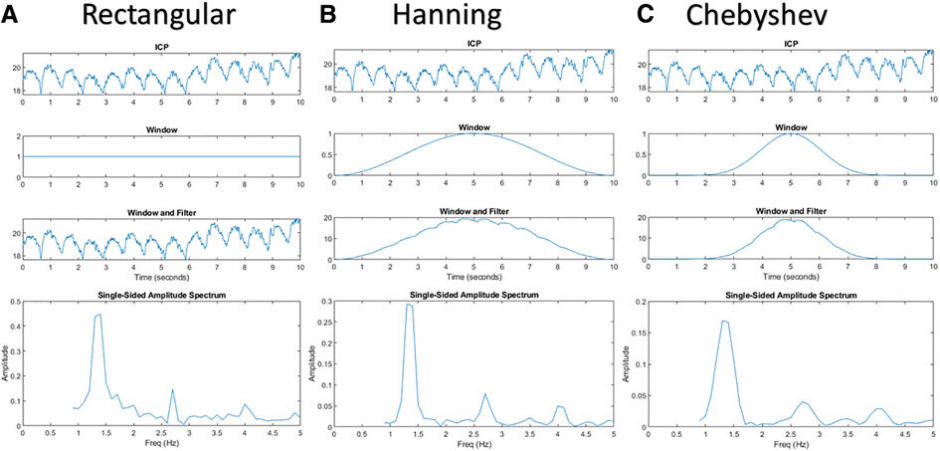
Different DFT windowing over ICP signals. There are three window types: rectangular (**A**), Hanning (**B**), and Chebyshev (**C**). Three different windowing methods utilized to assess AMP are compared. Note the amplitude and overall small differences in the single-sided amplitude spectrum. AMP, pulse amplitude of ICP; DFT, discrete Fourier transform; Freq, frequency; ICP, intracranial pressure.

**FIG. 2.**
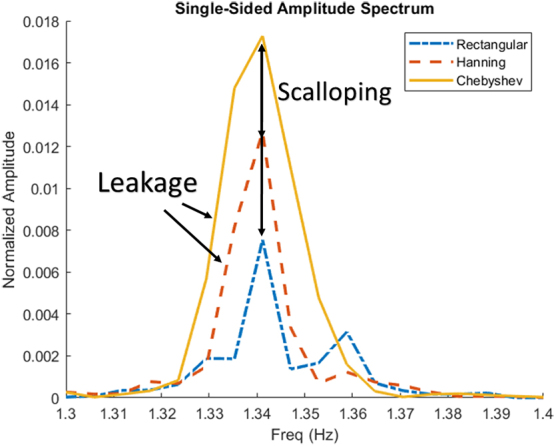
Examples of spectral and amplitude errors in ICP signal. Figure demonstrates the slight differences in the different windowing methods on ICP found AMP, note that the power is normalized over the data. Chebyshev has the best amplitude resolution and rectangular has the best frequency resolution. AMP, pulse amplitude of ICP; Freq, frequency; ICP, intracranial pressure.

For the 100 patients, we calculated AMP over a 10-sec window using a rectangular (best window for frequency resolution), Hanning (most common and a balance between the other two), and Chebyshev (chosen as one of the best amplitude resolution methods in ICM+) window types. Examples of this can be seen in Figures 1 and 2, which highlight some general differences in these methods. AMP values were compared within each patient using the Wilcoxon signed-ranked test between the rectangular window and the other two methods.

Using the previously described AMPs, PAx was found for each method. Then, the Wilcoxon signed-ranked test between the PRx and PAx methods, as well as the Wilcoxon signed-ranked test between the rectangle PAx versus Hanning/Chebyshev PAx, was found. We also found the percentage of time that PRx and PAx measures were above key thresholds of impaired CVR (>0, >0.25, and >0.3).

Additionally, the average CPPopt value for each of the CVR measures and the Wilcoxon signed-ranked test between them were also found.

To help better visualize the relationships, we also leveraged histogram plots of the median values for each comparison.

Finally, given that previous literature suggests a potential impact of biological sex on cerebral physiology post-TBI,^23–26^ we performed a subsequent subanalysis based on male versus female.

## Results

### Patient population characteristics

From this database, 100 TBI patients were included in this study. Average age was 44 years, with 83 being males, which is in keeping with past studies of this population. Table 2 summarizes the demographic data.

**Table 2.** Demographic Information

Variable	Amount/median (interquartile range)
No. of patients	100
Age (years)	42 (27–57)
Sex (male)	83 (83%)
Best admission GCS	7 (4–8)
Duration of recording (days)	3 (1–6)
	
Pupils	
Pupil bilateral reactive	63 (63%)
Unilateral unreactive	22 (22%)
Bilateral unreactive	13 (13%)
	
Marshall CT score	
VI	0 (0%)
V	46 (46%)
IV	20 (20%)
III	31 (31%)
II	3 (3%)
I	0 (0%)

GCS, Glasgow Coma Scale; CT, computed tomography.

### Pulse amplitude of intracranial pressure derivation: impact of windowing

Figures 1 and 2 visually demonstrate the differences between the rectangular, Hanning, and Chebyshev windowing methods. For Figure 1, note that the overall amplitude is scaled differently for the single-sided amplitude spectrum and how the spectral leakage is slightly different in each method. For Figure 2, the amplitude has been normalized over the whole signal, from which the key difference in each method can be seen. Rectangular and Hanning have scalloped amplitude loss compared to Chebyshev, and, inversely, Chebyshev and Hanning have frequency leakage compared to the rectangular window.

**FIG. 3.**
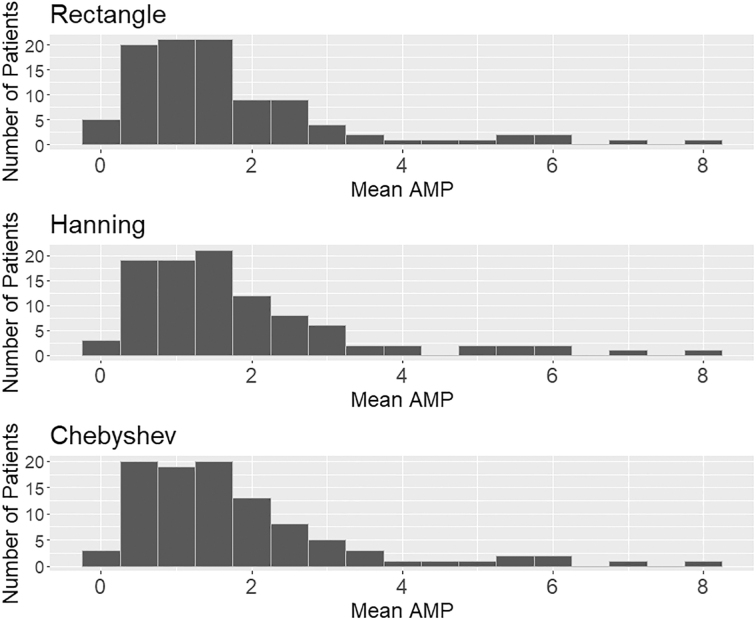
Distribution of average patient AMP. Histogram distribution of AMPs for different patients using different DFT windowing types, note that they are similar though there are slight differences. AMP, pulse amplitude of ICP; DFT, discrete Fourier transform; ICP, intracranial pressure.

Figure 3 shows the histogram distribution of the per patient AMP data, though most patients had significantly similar results. There were some outliers in this relationship where the different AMP method used would result in slightly different individual patient results (as can be seen in Supplementary Appendix Table SC1).

### Cerebrovascular reactivity

When comparing PRx and PAx measures, they were similar, though there were some examples of outliers that did not match (see Supplementary Appendix Table SC2). This was also observed in the relationship between the individual PAx measures, thus highlighting that there were small differences. However, over the whole population, and when measuring the percentage of time over key thresholds as shown in Table 3, the windowing type did not grossly impact the results of CVR.

**Table 3.** Percent Time Over Thresholds

	PRx	PAx rectangular		PAx Hanning		PAx Chebyshev	
	Median (IQR)	Median (IQR)	*p *value	Median (IQR)	*p *value	Median (IQR)	*p *value
% time >0.0	68.2 (53.4–84.8)	51.1 (35.7–69.4)	**<0.0001**	54.5 (37.1–70.5)	**<0.0001**	53.4 (36.4–68.8)	**<0.0001**
% time >0.25	41.1 (27.6–60.0)	24.4 (12.5–42.0)	**<0.0001**	26.2 (14.5–44.1)	**<0.0001**	26.2 (14.0–43.5)	**<0.0001**
% time >0.3	36.1 (23.1–53.7)	19.3 (10.4–37.4)	**<0.0001**	21.6 (11.7–38.5)	**<0.0001**	21.1 (11.1–37.0)	**<0.0001**

*p* value between PRx and given PAx value. Highlighted *p* values <0.05.

AMP, pulse amplitude; IQR, interquartile range; PAx, pulse amplitude index; PRx, pressure reactivity index.

### Optimal cerebral perfusion pressure

**FIG. 4.**
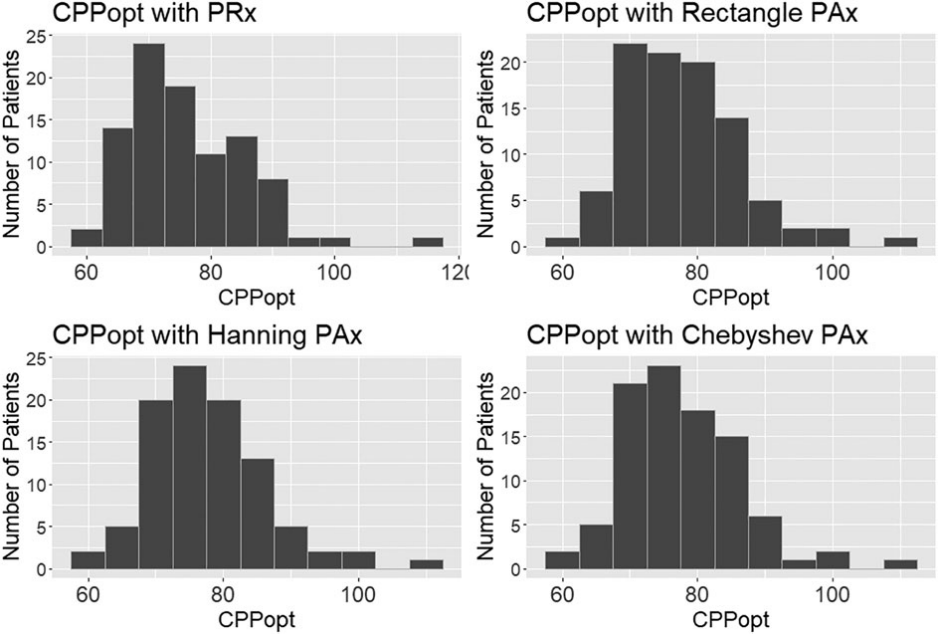
Distribution of average patient CPPopt. Histogram distributions of CPPopts for different patients using different DFT windowing types; note that they are similar, though there are slight differences. CPPopt, optimal cerebral perfusion pressure; DFT, discrete Fourier transform.

Like the other methods, CPPopt has only minor differences; however, the variation was minimal being a CPP of ±5 mm Hg. Like CVR and AMP, on an individual patient-by-patient comparison, there were some outliers to whom CPPopt did not match between the methods (see Supplementary Appendix Table SC3). However, over the entire population and over the whole data, they were sufficiently similar (see Fig. 4).

### Dichotomization on sex

The overall results were similar to the full data for both sexes. Given this similarity with the full database, these results will not be shown directly in this article and are only mentioned here for completeness.

## Discussion

We compared different windowing techniques used to find the spectral domain of AMP and AMP-based measurements of cerebral assessment. To this end, we highlighted the practical differences within different windowing methods as well as the associated errors. Focusing on the window type, we documented other miscellaneous errors and highlighted the key features of different windows utilized in data analysis.

For practical applications, the sampling rate and window size will impact the resulting spectral leakage, scalloping, and magnitude modification. With these three errors, the resulting amplitude magnitude can vary significantly between measurements. Although standardizing the window size will account for magnitude modification, because of the transient nature of pathophysiological phenomena, it is difficult to select a sampling rate and window width location that perfectly encapsulate the full period of the desired waveform. Therefore, in real-world applications of a DFT, choosing the window type that gives more precise results for a given calculation is recommended.

As can be seen in Figure 1 and 2, the different windowing methods have slightly different results, which have marginal trade-offs between frequency or amplitude accuracy. In an individual AMP-by-AMP or patient-by-patient calculation, the DFT windowing type did impact the result; however, when finding the overall AMP, CVR, or CPPopt, the differences between the DFT windows had a minimal impact. Moreover, measures like PRx and PAx already have a high variance in their response, though when comparing their percentage of time over key thresholds, the variance tended to resolve out. Likewise, for the DFT windows of PAx, the percentage of time over key thresholds were found to be similar. Thus, for general large aggregates of data, the type of DFT window utilized is largely indiscriminate.

For practical applications of a DFT, often no windowing techniques are used (i.e., rectangular window) given that there are no extra computational steps required. Despite this, as data becomes more specialized and individualized, the need to use windowing for improved performance should be considered. As an example, heart-rate variability and vasogenic response in currently outlined methodologies do not use any windowing methods.^1,27–31^ Both of these physiological measurements evaluate the overall amplitude or power from a range of frequency components. Thus, these methods do not require the frequency precision from rectangular windows, but would benefit from the amplitude accuracy of Chebyshev or like windows.

In past literature applications of a DFT that used the rectangular window, the application of a windowing method would have a limited impact given that past work is primarily focused on general physiological response.^1,27–31^ However, the amplitude accuracy lost in rectangular windows may play a more significant role in physiological assessment as more momentary and personalized assessment becomes important. Moreover, using windowing methods that give more amplitude accuracy would allow for more minor and acute amplitude changes to be assessed and should be recommended for further methods where amplitude response is assessed over a wide range of frequency components.

Though it is always recommended to utilize a window that best encapsulates the data one is trying to find, there were some noticeable and, in some measurements, quite apparent differences within the overall results, as highlighted in Figure 2. For methods where frequency resolution is the most important, a rectangular window should be used; inversely, when amplitude accuracy is the most important, a Chebyshev or similar window type should be used. A balance between both windows is something like the Hanning window. As demonstrated here, often in physiological measures focusing on grand mean changes of physiological phenomenon DFT windowing has a limited impact on the overall calculation.

## Conclusion

With the application of a DFT, the spectral analysis of continuous physiological data can be performed. To apply a DFT in a reasonable manner, the desired data must be bound by a windowing method. From this, windowing errors can be introduced, though these errors can be mitigated depending on the windowing method used. Though in large aggregates of data or percentage of time over thresholds the type of windowing method had a negligible impact on the resulting calculations, for certain patients or individual outliers there were small differences in the resulting calculations. Thus, it is always best to pick the window that is most applicable to the desired focus of the analysis. When frequency accuracy is important, a rectangular window should be used, whereas for amplitude accuracy a Chebyshev or similar window should be considered.

## Supplementary Material

Supplemental data

## Supplementary Material

Supplementary Appendix SA

Supplementary Appendix SB

Supplementary Appendix SC

## Abbreviations Used

AMP: pulse amplitude of intracranial pressure
ABP: arterial blood pressure
CPP: cerebral perfusion pressure
CPPopt: optimal cerebral perfusion pressure
CVR: cerebrovascular reactivity
DFT: discrete Fourier transform
FT: Fourier transform
ICP: intracranial pressure
PAx: pulse amplitude index
PRx: pulse reactivity index
TBI: traumatic brain injury
