# Supplementary material for: Discrete Fourier Transform Windowing Techniques for Cerebral Physiological Research in Neural Injury: A Practical Demonstration
Source: Neurotrauma Rep. 2023 Jun 22;4(1):410–9. doi: 10.1089/neur.2022.0079 (PMC10288301; doi:10.1089/neur.2022.0079)
Supplement: Supplemental data [file Suppl_Appendix.docx]

Contents

[Appendix A. Fourier Transform (FT) 2](#_Toc126296595)

[Figure A1. DFT Example 2](#_Toc126296596)

[Appendix A1. DFT Windowing 2](#_Toc126296597)

[Figure A2. Different Window Types 3](#_Toc126296598)

[Figure A3. Windowing Examples and Key Differences 4](#_Toc126296599)

[Appendix A1 Spectral Leakage 5](#_Toc126296600)

[Figure A4. Scalloping and Spectral Leakage 5](#_Toc126296601)

[Appendix A2 Scalloping 6](#_Toc126296602)

[Appendix A3 Magnitude Modification 6](#_Toc126296603)

[Appendix A4 Bandwidth Noise 6](#_Toc126296604)

[Appendix B. Practical Considerations 6](#_Toc126296605)

[Appendix B1 Artifact data 6](#_Toc126296606)

[Appendix B2 Windowing Size and Sample rate 6](#_Toc126296607)

[Appendix C. Pulse amplitude index (PAx) and Cerebrovascular reactivity 7](#_Toc126296608)

[Table C1. Windowing Differences on AMP 7](#_Toc126296609)

[Table C2. Windowing Differences on PAx 11](#_Toc126296610)

[Table C3. Windowing Differences on CPPopt 14](#_Toc126296611)

[References 17](#_Toc126296612)

# Appendix A. Fourier Transform (FT)

FT was initially used to describe the relationship of heat diffusion between two metal plates (how heat reaches thermal equilibrium)^1^. Through this observation, Fourier deduced that any continuous signal can be created by a series sum of sine waves^1^. Since the advent of computer processing, this methodology has been adopted to a variety of applications from computational analytics through to physiological data analysis. The more practical application in computational methodology is a Discrete Fourier Transform (DFT) which takes the main concept behind a FT (representing the continuous signal by a sum of sine waves) and reducing it into a discrete and digitally approachable manner. Figure A1 shows an example of DFT methodology on a simple sine wave, demonstrating the key benefits of representing continuous physiology in the frequency domain (it is easier to identify the key frequencies as well as the magnitude of said frequencies in oscillatory human physiology). Note in the practical application of a DFT, the data needs to be isolated to a window of time, as it is impossible to collect an infinite signal and often the desire is to look at localized time phenomena.

### Figure A1. DFT Example

**
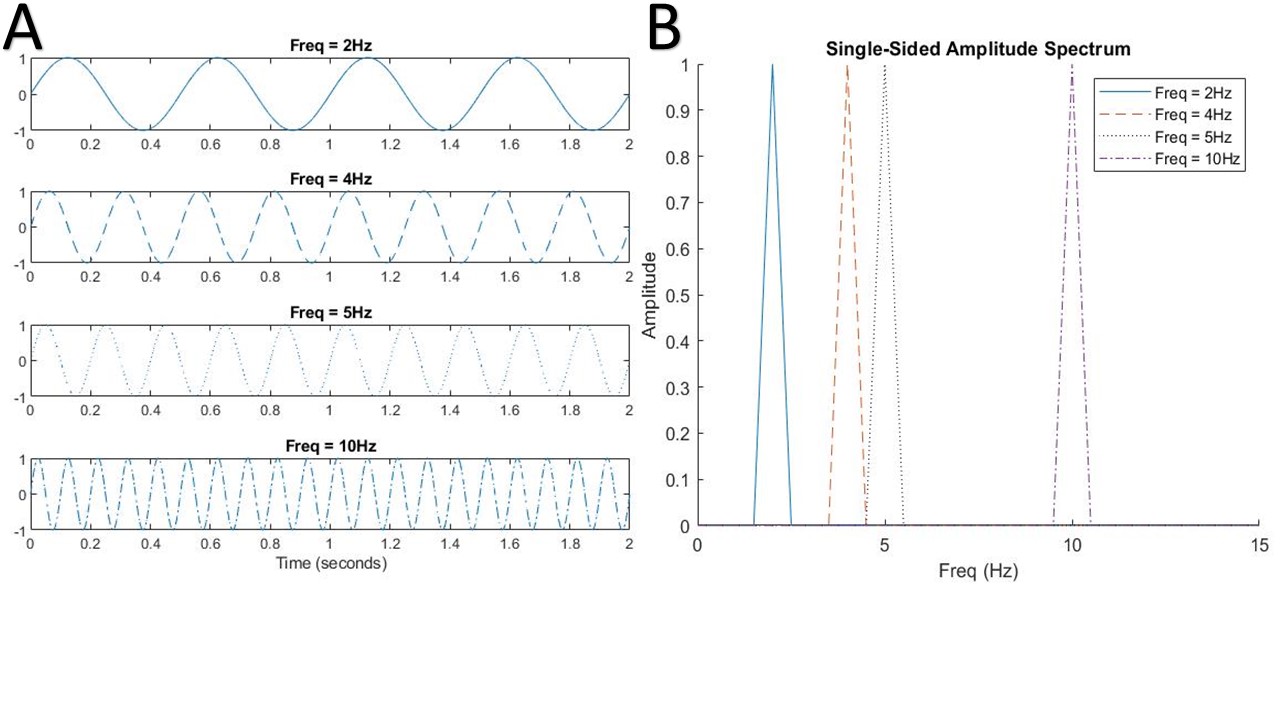
**

*‘A’ are examples of sine waves at different frequencies, and ‘B’ are the four signals transformed using a DFT to the spectral/frequency domain (i.e., they are represented by a sinewave’s amplitude and frequency). DFT, discrete Fourier transform; Freq, frequency; Hz, hertz.*

## Appendix A1. DFT Windowing

Windowing is the process that isolates a small subset of data over a localized time, for most applications it involves truncating the data outside a range and conforming the data within the range to a function. Different types of windows are shown in Appendix B.

### Figure A2. Different Window Types

**
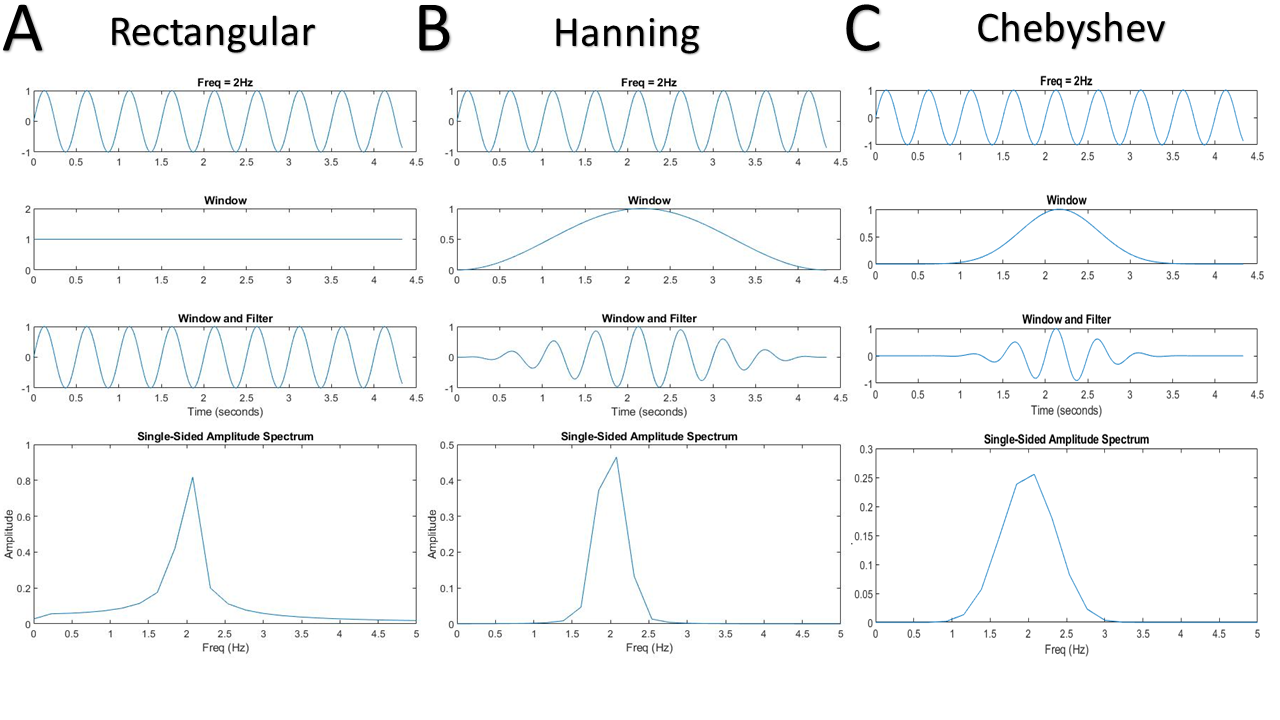
***There are 3 window types: Rectangular (‘A’), Hanning(‘B’), and Chebyshev(‘C’). In each, notice that the window is different as well as the resulting single sided spectral analysis. DFT, discrete Fourier transform; Freq, frequency; Hz, hertz.*

Through this windowing, there are many inaccuracies that occur including spectral leakage, scalloping, magnitude modification, and bandwidth noise (which will be discussed later). As a simple overview, in the context of time-frequency analysis, there are two things to remember: the size of the window and the window type used.

The wider the analysis window, the better the spectral resolution and the worse the temporal resolution. Thus, to detect transient events, a shorter window that encompasses the time of the transient event is ideal.

Figure A2 gives a visual demonstration of different types of windows. Any window type is characterized by two quantities: the dynamic range and the resolution. The dynamic range refers to the ability of the resulting spectral domain to differentiate between the components of neighboring frequencies (different sine waves). Resolution refers to the accuracy in the resulting magnitude for the collected frequencies (the overall amplitude given to each frequency component). In Figure A3, you can see a DFT of windowing functions, showing a main lobe and side lobes in the spectral domain.

For practical applications, the narrower the main lobe, the better the ability of the window to distinguish between closely placed frequency components. The lower the amplitudes of the side lobes, the better the window's ability to distinguish between frequency components’ amplitudes. Unfortunately, windows that are good for one of these objectives tend to be worse for the other. The Rectangular window has the best resolution (the main lobe is narrow), but it has a bad dynamic range (the side lobes have a high magnitude). Therefore, in several practical applications, other windows are preferred, such as Hanning and Hamming, as they better compromise between resolution and dynamic range.

### Figure A3. Windowing Examples and Key Differences

**
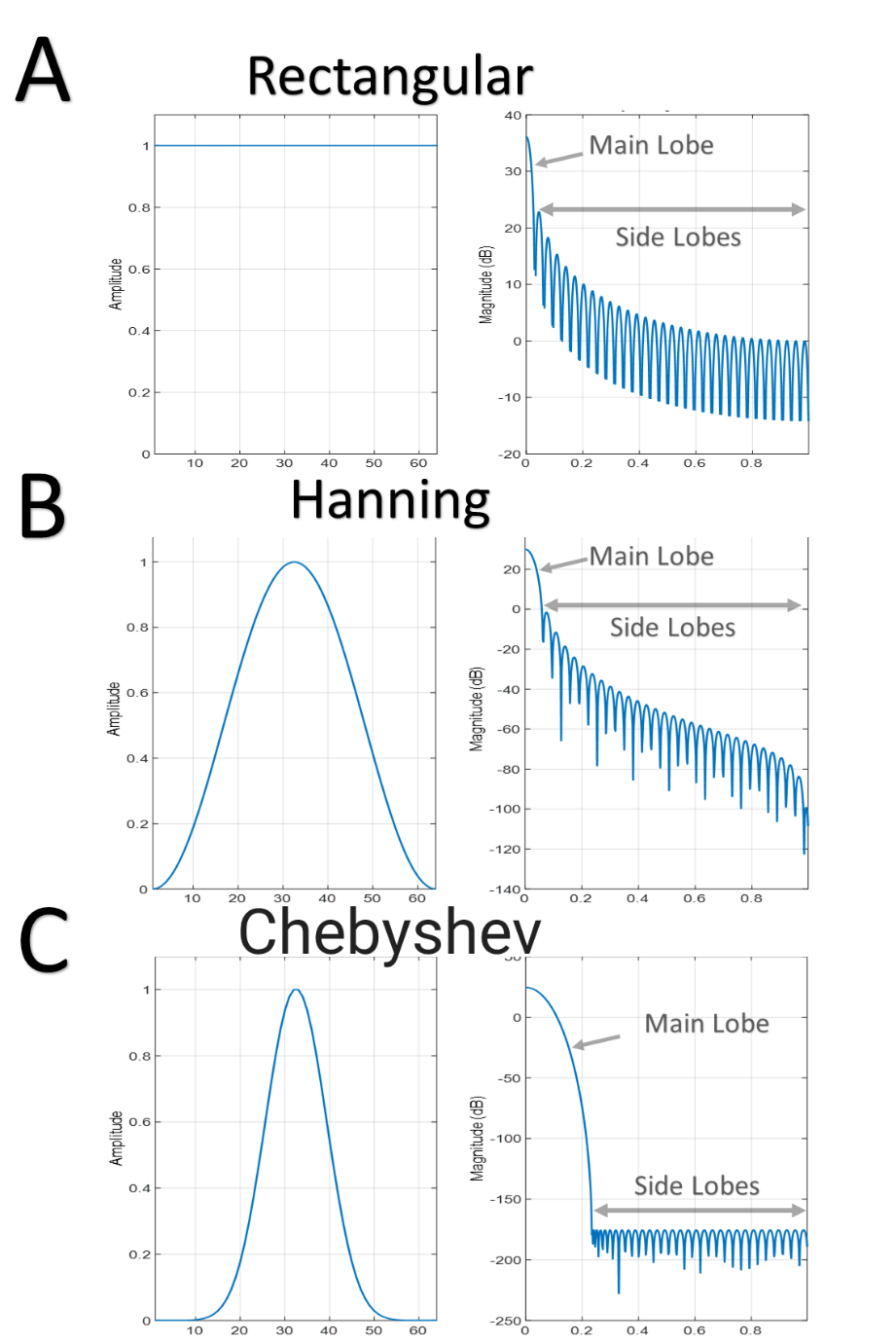
**

*‘A’ is a rectangular window, ‘B’ is a Hanning and ‘C’ is a Chebyshev. The left column demonstrates the time domain and the right demonstrates the spectral domain. Note the difference in main lobe width as well as the ratio between the magnitude of the main and side lobes. From this, Chebyshev can be said to have the best amplitude resolution (magnitude difference between the main and side lobes) and Rectangular can be said to have the best frequency resolution (smallest main lobe width). dB, decibels.*

## Appendix A1 Spectral Leakage

When signal energy from one frequency component leaks into another frequency component in a DFT, the accuracy of the original signal is altered (this can be seen in Figure A4). This is called Spectral Leakage. In application, the spectral leakage will degrade the amplitude accuracy and frequency resolution of the spectral domain.

### Figure A4. Scalloping and Spectral Leakage

**
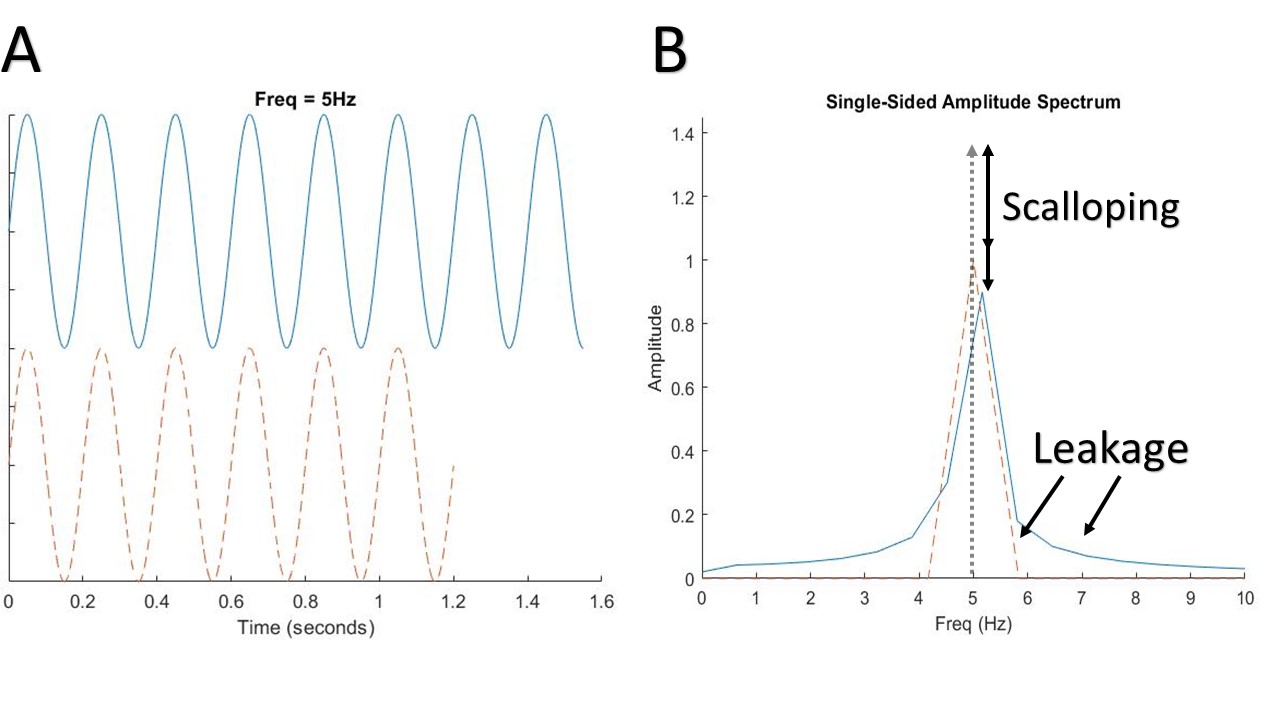
**

*‘A’ is the time domain of 2 signals and ‘B’ is the spectral domain. This is a figure of the time and spectral domain for one signal with different windowing lengths. The longer window is not a factor of the fundamental frequency which results in more spectral loss and scalloping. From the differences in amplitudes, scalloping can be visualized with spectral leakage demonstrated in both as compared to the idealized black hashed arrow at 5Hz (i.e. ideally there would only be one frequency component at 5Hz). DFT, discrete Fourier transform; Freq, frequency; Hz, hertz.*

## Appendix A2 Scalloping

The reduction in energy from the main lobe is called scalloping (this can be seen in Figure A4), which is caused by the frequency components being discrete. All computational data is discrete meaning it is individualized, countable and divisible. In the application of a DFT, the desired frequency component cannot be perfectly isolated and thus some of one frequency component will be represented in two or more components. To avoid this, prearranged choices of sampling rate and a window length that results in an integer number of cycles within the desired frequency should be used.

## Appendix A3 Magnitude Modification

A DFT results from adding signal samples, so the longer the signal is, the larger the sum of the data. In order to get a frequency component magnitude, a DFT scales the frequency components according to the number of signal components and total energy. Thus, a DFT, by default, does not normalize the power and therefore the spectral component’s power is dependent on the length of window analyzed. To account for this, often a DFT will be normalized. Normalization is a process to reduce or conform the data to a standardized measure. Through this normalization, the resulting magnitude scales will have uniformity with respects to the data. Therefore, the overall change of the magnitude will be sacrificed to have linearity within the resulting spectral domain (this means that the true amplitude is sacrificed for better internal spectral domain coherency).

## Appendix A4 Bandwidth Noise

Bandwidth noise describes the error that occurs due to white noise of the signal source (the impact that natural interference has on physiology recordings). It is defined as the size of the difference in overall amplitude between the signal itself and the noise in the recording. Depending on the type of window used and the time of the window, this can be negligible in identifying desired physiology.

# Appendix B. Practical Considerations

## Appendix B1 Artifact data

Though it is always best to have a clean, artifact free signal, this is not always possible. In such cases, it is often advantageous to manually inspect the data for egregious errors. However, in relation to DFT, such errors may have a limited impact on the desired results.

For example, if the artifact is a one-time phenomenon over the window analyzed, it will appear in ultra-high frequencies, which are often outside the area of interest. Moreover, if the artifact is reoccurring and stable, it can also be removed in the Spectral domain. Though it should be advised that such artifacts will influence the amplitude of the resulting spectral analysis (the overall magnitude will be changed but the linear relationship will remain relatively the same).

## Appendix B2 Windowing Size and Sample rate

As discussed in spectral loss, choosing an optimal sampling rate can help reduce this error by selecting a rate whose frequency component is a factor of the sampling rate. Moreover, at a minimum the sampling rate should be chosen to be at least 4 to 10 time that of the desired frequency (note this assumes that the signal is a perfect sinewave).^2^

In combination with this, the window size can play a role in the accuracy of the spectral domain. Figure 4 shows an example of the same signal and sampling rate with different window sizes. Like the sampling rate, if the desired frequency component is known, choosing a window size that is a factor of this frequency component is best. However, most applications of a DFT require the window length to be at least 0.5 the length of the slowest component assessed.

# Appendix C. Pulse amplitude index (PAx) and Cerebrovascular reactivity

This methodology is taken from our previous studies with all patients having high-frequency digital signals recorded throughout their ICU stay.^3–6^ Arterial blood pressure (ABP) was obtained through radial or femoral arterial lines connected to pressure transducers (Baxter Healthcare Corp. CardioVascular Group, Irvine, CA, or similar devices). Intracranial blood pressure (ICP) was acquired via an intra-parenchymal strain gauge probe (Codman ICP MicroSensor; Codman & Shurtlef Inc., Raynham, MA). These signals were captured simultaneously and digitized via an A/D converter (DT9804; Data Translation, Marlboro, MA), sampled at a frequency of 100 Hertz (Hz) or higher, using the Intensive Care Monitoring (ICM+) software (Cambridge Enterprise Ltd, Cambridge, UK, http:// icmplus.neurosurg.cam.ac.uk). All signal artifacts were removed using manual methods before further processing and analysis. Local research ethics board approval at the University of Manitoba is in place for all aspects of this database (H2017:181 and H2017:188).

Mean arterial blood pressure (MAP) and ICP were calculated as the mean value over a 10 second window, from the ABP and ICP waves. CPP was derived as MAP - ICP. PAx methodology is taken from the methods outlined by Aries et al. and Zeiler et al. by measuring the pulse amplitude of an ICP wave (AMP) over a 10 second window and comparing it with MAP.^7–11^ The PRx is the most common method of cerebrovascular reactivity and is determined through a Pearson’s correlation between ICP and ABP.^12–14^ Finally optimal cerebral perfusion pressure (CPPopt) was determined in individual patients through the use of the OPT Flex methodology.^12,15–17^ For this method, PRx/PAx values were divided and averaged into CPP bins spanning 5 mmHg. Then an automatic curve fitting method was applied to the binned CPP data to determine the CPP value with the lowest associated PRx/PAx value.

For the 100 patients we calculated AMP over a 10 second window using a Rectangular, Hanning, and Chebyshev (chosen as one of the best amplitude resolution methods in ICM+) window types. We also found the PRx and PAx for the previously described AMPs. For all test an alpha of significance was set to 0.05 with no tests used to adjust for multiple comparison since the data is used given the high variability in p values. Table C1 and C2 demonstrate the various results from these windows and the Wilcox signed ranked test between the Rectangular window and the other two methods. Finally, table C3 is the average CPPopt value for each of the cerebrovascular reactivity measures and the Wilcox signed ranked test between the rectangular window and PRx, and the Rectangular window and the other two methods.

## Table C1. Windowing Differences on AMP

| **Patient** | **AMP Rectangular** | **AMP Hanning** | | **AMP Chebyshev** | |
| --- | --- | --- | --- | --- | --- |
|  | **Median (IQR)** | **Median (IQR)** | **Rectangular and Hanning p value** | **Median (IQR)** | **Rectangular and Chebyshev p value** |
| 1 | 3.43 (2.54-4.46) | 3.71 (2.74-4.83) | **<0.0001** | 3.58 (2.66-4.66) | **<0.0001** |
| 2 | 2.68 (1.68-3.83) | 2.86 (1.82-3.98) | **<0.0001** | 2.77 (1.75-3.9) | **<0.0001** |
| 3 | 1.43 (1.04-1.81) | 1.51 (1.1-1.91) | **<0.0001** | 1.48 (1.08-1.86) | **<0.0001** |
| 4 | 3.2 (2.18-4.63) | 3.26 (2.21-4.71) | **<0.0001** | 3.24 (2.2-4.68) | **0.000805** |
| 5 | 2.92 (1.25-5.12) | 3.05 (1.59-5.29) | **<0.0001** | 3 (1.5-5.22) | **<0.0001** |
| 6 | 1.7 (1.25-2.44) | 1.87 (1.41-2.69) | **<0.0001** | 1.8 (1.34-2.59) | **<0.0001** |
| 7 | 2.28 (1.74-3.17) | 2.62 (2.02-3.65) | **<0.0001** | 2.45 (1.9-3.42) | **<0.0001** |
| 8 | 0.119 (0.0504-0.256) | 0.125 (0.0519-0.279) | **<0.0001** | 0.123 (0.0516-0.27) | **0.000516** |
| 9 | 1 (0.84-1.18) | 1.2 (0.996-1.37) | **<0.0001** | 1.11 (0.948-1.3) | **<0.0001** |
| 10 | 1.24 (1.06-1.49) | 1.26 (1.08-1.52) | **<0.0001** | 1.25 (1.07-1.51) | **0.000224** |
| 11 | 1.45 (1.07-1.71) | 1.53 (1.17-1.81) | **<0.0001** | 1.5 (1.14-1.76) | **<0.0001** |
| 12 | 2.67 (2.26-3.26) | 2.75 (2.34-3.44) | **<0.0001** | 2.71 (2.31-3.35) | **<0.0001** |
| 13 | 8.73 (7.21-9.74) | 8.8 (7.26-9.78) | **0.008252** | 8.76 (7.24-9.76) | 0.153361 |
| 14 | 2.64 (1.9-3.54) | 2.92 (2.17-3.8) | **<0.0001** | 2.81 (2.07-3.69) | **<0.0001** |
| 15 | 2.28 (1.85-2.72) | 2.41 (1.98-2.9) | **<0.0001** | 2.35 (1.92-2.81) | **<0.0001** |
| 16 | 7.54 (6.08-8.13) | 7.57 (6.1-8.15) | 0.096778 | 7.55 (6.09-8.14) | 0.339399 |
| 17 | 3.24 (1.55-6.44) | 3.31 (1.59-6.61) | **<0.0001** | 3.28 (1.57-6.54) | **0.013333** |
| 18 | 0.365 (0.198-0.598) | 0.4 (0.231-0.632) | **<0.0001** | 0.386 (0.222-0.617) | **<0.0001** |
| 19 | 0.83 (0.521-1.22) | 0.89 (0.624-1.36) | **<0.0001** | 0.872 (0.596-1.3) | **<0.0001** |
| 20 | 0.882 (0.617-1.12) | 0.945 (0.706-1.18) | **<0.0001** | 0.92 (0.676-1.15) | **<0.0001** |
| 21 | 0.824 (0.573-1.47) | 1.89 (1.47-2.31) | **<0.0001** | 1.78 (1.38-2.17) | **<0.0001** |
| 22 | 5.62 (3.09-7.6) | 5.72 (3.4-7.68) | **<0.0001** | 5.68 (3.37-7.64) | **<0.0001** |
| 23 | 2.28 (1.84-2.79) | 2.51 (2.08-3.06) | **<0.0001** | 2.4 (1.97-2.93) | **<0.0001** |
| 24 | 1.43 (0.873-2.88) | 1.56 (0.914-3.22) | **0.018361** | 1.52 (0.901-3.08) | 0.138555 |
| 25 | 0.852 (0.611-1.63) | 0.981 (0.681-1.94) | **<0.0001** | 0.926 (0.653-1.82) | **<0.0001** |
| 26 | 0.576 (0.45-0.71) | 0.665 (0.535-0.798) | **<0.0001** | 0.625 (0.502-0.758) | **<0.0001** |
| 27 | 1.28 (0.943-1.68) | 1.33 (1-1.75) | **<0.0001** | 1.31 (0.978-1.72) | **<0.0001** |
| 28 | 0.975 (0.702-1.48) | 1.07 (0.767-1.63) | **<0.0001** | 1.03 (0.739-1.56) | **<0.0001** |
| 29 | 1.82 (1.17-2.56) | 1.9 (1.25-2.66) | **<0.0001** | 1.86 (1.22-2.61) | **<0.0001** |
| 30 | 1.62 (1.2-2.15) | 1.81 (1.36-2.43) | **<0.0001** | 1.72 (1.28-2.3) | **<0.0001** |
| 31 | 0.842 (0.643-1.13) | 0.934 (0.732-1.24) | **<0.0001** | 0.895 (0.696-1.19) | **<0.0001** |
| 32 | 1.27 (1.07-1.54) | 1.32 (1.14-1.58) | **<0.0001** | 1.29 (1.11-1.56) | **<0.0001** |
| 33 | 1.41 (1.04-1.78) | 1.56 (1.19-1.9) | **<0.0001** | 1.49 (1.13-1.85) | **<0.0001** |
| 34 | 0.223 (0.169-0.284) | 0.262 (0.208-0.32) | **<0.0001** | 0.248 (0.197-0.306) | **<0.0001** |
| 35 | 2.13 (1.56-3.12) | 2.39 (1.8-3.36) | **<0.0001** | 2.27 (1.69-3.25) | **<0.0001** |
| 36 | 1.53 (1.14-1.8) | 1.64 (1.3-1.92) | **<0.0001** | 1.59 (1.23-1.86) | **<0.0001** |
| 37 | 6.06 (5.08-7.18) | 6.12 (5.14-7.26) | **<0.0001** | 6.09 (5.11-7.22) | **0.103908** |
| 38 | 0.772 (0.641-1.35) | 0.802 (0.664-1.43) | **<0.0001** | 0.788 (0.656-1.4) | **<0.0001** |
| 39 | 0.492 (0.382-0.824) | 0.549 (0.421-0.969) | **<0.0001** | 0.525 (0.402-0.91) | **<0.0001** |
| 40 | 0.506 (0.348-0.721) | 0.572 (0.41-0.814) | **<0.0001** | 0.541 (0.386-0.77) | **<0.0001** |
| 41 | 1.22 (0.957-1.51) | 1.4 (1.12-1.65) | **<0.0001** | 1.32 (1.06-1.58) | **<0.0001** |
| 42 | 0.692 (0.341-0.953) | 0.785 (0.37-1.08) | **<0.0001** | 0.739 (0.359-1.01) | **<0.0001** |
| 43 | 0.892 (0.705-1.12) | 0.987 (0.776-1.21) | **<0.0001** | 0.938 (0.745-1.16) | **<0.0001** |
| 44 | 0.505 (0.226-0.738) | 0.561 (0.246-0.787) | **<0.0001** | 0.537 (0.24-0.764) | **<0.0001** |
| 45 | 1.17 (0.969-1.31) | 1.24 (1.1-1.39) | **<0.0001** | 1.21 (1.04-1.36) | **<0.0001** |
| 46 | 0.48 (0.352-0.707) | 0.568 (0.411-0.822) | **<0.0001** | 0.539 (0.396-0.788) | **<0.0001** |
| 47 | 0.133 (0.112-0.22) | 0.136 (0.121-0.195) | **0.026663** | 0.135 (0.118-0.202) | 0.161894 |
| 48 | 1.89 (1.24-3.45) | 1.9 (1.24-3.48) | **<0.0001** | 1.9 (1.24-3.46) | 0.137217 |
| 49 | 2.2 (1.12-2.52) | 2.24 (1.37-2.56) | **<0.0001** | 2.22 (1.29-2.54) | **<0.0001** |
| 50 | 0.446 (0.294-0.572) | 0.475 (0.318-0.592) | **<0.0001** | 0.463 (0.309-0.583) | **<0.0001** |
| 51 | 2.47 (2.01-2.95) | 2.61 (2.16-3.13) | **<0.0001** | 2.55 (2.09-3.04) | **<0.0001** |
| 52 | 0.35 (0.295-0.38) | 0.36 (0.309-0.389) | **<0.0001** | 0.356 (0.306-0.385) | **<0.0001** |
| 53 | 1.05 (0.805-1.21) | 1.07 (0.844-1.23) | **<0.0001** | 1.06 (0.828-1.22) | **0.008741** |
| 54 | 1.51 (1.22-1.89) | 1.64 (1.34-2.1) | **<0.0001** | 1.58 (1.29-2) | **<0.0001** |
| 55 | 0.765 (0.573-1.08) | 0.892 (0.627-1.2) | **<0.0001** | 0.839 (0.605-1.14) | **<0.0001** |
| 56 | 1.43 (1.11-1.74) | 1.52 (1.2-1.85) | **<0.0001** | 1.47 (1.17-1.79) | **<0.0001** |
| 57 | 0.499 (0.339-0.715) | 0.554 (0.376-0.781) | **<0.0001** | 0.532 (0.361-0.751) | **<0.0001** |
| 58 | 1.94 (1.33-2.78) | 2.15 (1.47-3.11) | **<0.0001** | 2.05 (1.41-2.96) | **<0.0001** |
| 59 | 1.31 (0.898-1.78) | 1.43 (0.956-1.97) | **<0.0001** | 1.37 (0.931-1.88) | **<0.0001** |
| 60 | 2.24 (1.73-2.81) | 2.39 (1.87-2.94) | **<0.0001** | 2.33 (1.81-2.88) | **<0.0001** |
| 61 | 0.406 (0.351-0.528) | 0.437 (0.384-0.596) | **<0.0001** | 0.424 (0.372-0.575) | **<0.0001** |
| 62 | 5.16 (4.19-6.52) | 5.22 (4.28-6.58) | **<0.0001** | 5.19 (4.24-6.55) | **<0.0001** |
| 63 | 4.43 (2.98-5.79) | 4.62 (3.16-6.01) | **<0.0001** | 4.52 (3.1-5.9) | **0.004858** |
| 64 | 0.712 (0.487-1) | 0.789 (0.548-1.09) | **<0.0001** | 0.754 (0.524-1.05) | **<0.0001** |
| 65 | 1.27 (1.04-1.52) | 1.36 (1.16-1.61) | **<0.0001** | 1.32 (1.12-1.57) | **<0.0001** |
| 66 | 1.65 (0.913-2.56) | 1.73 (0.948-2.63) | **<0.0001** | 1.69 (0.936-2.6) | **0.008104** |
| 67 | 1.21 (0.89-1.74) | 1.35 (1.01-1.93) | **<0.0001** | 1.29 (0.961-1.84) | **<0.0001** |
| 68 | 1.57 (1.19-2.03) | 1.74 (1.35-2.17) | **<0.0001** | 1.66 (1.28-2.1) | **<0.0001** |
| 69 | 0.42 (0.338-0.499) | 0.457 (0.387-0.531) | **<0.0001** | 0.443 (0.369-0.517) | **<0.0001** |
| 70 | 1.23 (0.86-1.64) | 1.38 (0.973-1.81) | **<0.0001** | 1.31 (0.918-1.73) | **<0.0001** |
| 71 | 0.677 (0.557-0.813) | 0.7 (0.579-0.841) | **<0.0001** | 0.689 (0.57-0.828) | **<0.0001** |
| 72 | 0.343 (0.259-0.454) | 0.382 (0.301-0.508) | **<0.0001** | 0.367 (0.287-0.487) | **<0.0001** |
| 73 | 2.89 (2.21-3.51) | 3.04 (2.39-3.63) | **<0.0001** | 2.98 (2.32-3.57) | **<0.0001** |
| 74 | 0.793 (0.498-1.04) | 0.86 (0.543-1.09) | **<0.0001** | 0.833 (0.524-1.07) | **<0.0001** |
| 75 | 0.21 (0.0826-0.375) | 0.234 (0.0895-0.401) | **<0.0001** | 0.226 (0.088-0.39) | **<0.0001** |
| 76 | 0.294 (0.228-0.372) | 0.302 (0.232-0.379) | **<0.0001** | 0.298 (0.23-0.376) | **0.000698** |
| 77 | 1.7 (1.39-2.08) | 1.83 (1.51-2.24) | **<0.0001** | 1.78 (1.46-2.17) | **<0.0001** |
| 78 | 1.14 (0.953-1.4) | 1.19 (1.01-1.46) | **<0.0001** | 1.17 (0.986-1.43) | **<0.0001** |
| 79 | 0.66 (0.334-1.21) | 0.697 (0.375-1.26) | **<0.0001** | 0.68 (0.359-1.23) | **<0.0001** |
| 80 | 1.35 (1.03-1.68) | 1.51 (1.16-1.85) | **<0.0001** | 1.43 (1.1-1.77) | **<0.0001** |
| 81 | 0.972 (0.741-1.22) | 1.1 (0.834-1.35) | **<0.0001** | 1.05 (0.796-1.29) | **<0.0001** |
| 82 | 2.49 (1.78-3.24) | 2.74 (2.01-3.48) | **<0.0001** | 2.62 (1.91-3.36) | **<0.0001** |
| 83 | 1.54 (1.07-1.94) | 1.73 (1.23-2.12) | **<0.0001** | 1.64 (1.16-2.04) | **<0.0001** |
| 84 | 2.4 (1.81-2.71) | 2.47 (1.98-2.77) | **<0.0001** | 2.44 (1.91-2.74) | **<0.0001** |
| 85 | 5.05 (4.33-5.46) | 5.32 (4.88-5.61) | **<0.0001** | 5.18 (4.63-5.53) | **<0.0001** |
| 86 | 0.503 (0.337-0.7) | 0.548 (0.381-0.733) | **<0.0001** | 0.528 (0.363-0.718) | **<0.0001** |
| 87 | 0.354 (0.248-0.452) | 0.383 (0.264-0.474) | **<0.0001** | 0.373 (0.26-0.465) | **<0.0001** |
| 88 | 0.304 (0.212-0.423) | 0.341 (0.242-0.464) | **<0.0001** | 0.326 (0.23-0.445) | **<0.0001** |
| 89 | 1.87 (0.535-2.46) | 2.08 (1.08-2.48) | **<0.0001** | 2.05 (1.02-2.47) | **<0.0001** |
| 90 | 0.94 (0.661-1.27) | 1.05 (0.729-1.42) | **<0.0001** | 0.994 (0.697-1.35) | **<0.0001** |
| 91 | 1.2 (0.822-1.71) | 1.25 (0.872-1.76) | **<0.0001** | 1.23 (0.852-1.73) | **<0.0001** |
| 92 | 2.13 (1.78-2.38) | 2.16 (1.82-2.42) | **<0.0001** | 2.14 (1.8-2.4) | **<0.0001** |
| 93 | 0.823 (0.624-0.999) | 0.872 (0.703-1.05) | **<0.0001** | 0.851 (0.67-1.03) | **<0.0001** |
| 94 | 0.513 (0.335-0.955) | 0.644 (0.392-1.12) | **<0.0001** | 0.609 (0.384-1.05) | **<0.0001** |
| 95 | 1.62 (1.32-2.04) | 1.72 (1.43-2.17) | **<0.0001** | 1.68 (1.38-2.12) | **<0.0001** |
| 96 | 0.97 (0.746-1.36) | 1.08 (0.81-1.49) | **<0.0001** | 1.03 (0.783-1.43) | **<0.0001** |
| 97 | 0.368 (0.21-0.641) | 0.41 (0.242-0.722) | **<0.0001** | 0.393 (0.23-0.684) | **<0.0001** |
| 98 | 5.42 (4.09-6.83) | 5.65 (4.34-7.12) | **<0.0001** | 5.54 (4.24-6.98) | **<0.0001** |
| 99 | 0.778 (0.609-1.01) | 0.897 (0.713-1.15) | **<0.0001** | 0.846 (0.676-1.09) | **<0.0001** |
| 100 | 0.137 (0.0682-0.178) | 0.16 (0.0782-0.202) | **<0.0001** | 0.151 (0.0773-0.191) | **<0.0001** |
|  |  |  |  |  |  |

*Highlighted p values less than 0.05. AMP, pulse amplitude; IQR, interquartile range;*

## Table C2. Windowing Differences on PAx

| **Patient** | **PRx** | **PAx Rectangular** | | **PAx Hanning** | | | **PAx Chebyshev** | | |
| --- | --- | --- | --- | --- | --- | --- | --- | --- | --- |
|  | **Median (IQR)** | **Median (IQR)** | **PRx and PAx P value** | **Median (IQR)** | **PAx Rectangular and Hanning p value** | **PRx and PAx Hanning p value** | **Median (IQR)** | **PAx Rectangular and Chebyshev p value** | **PRx and PAx Hanning p value** |
| 1 | -0.0879 (-0.343-0.255) | -0.0051 (-0.27-0.302) | **<0.0001** | -0.00515 (-0.268-0.316) | 0.117796 | **<0.0001** | -0.0021 (-0.269-0.316) | 0.163757 | **<0.0001** |
| 2 | 0.0465 (-0.305-0.377) | -0.0624 (-0.349-0.206) | **<0.0001** | -0.0342 (-0.309-0.251) | **<0.0001** | **<0.0001** | -0.0476 (-0.326-0.236) | **<0.0001** | **<0.0001** |
| 3 | 0.111 (-0.11-0.417) | -0.112 (-0.313-0.0865) | **<0.0001** | -0.0822 (-0.282-0.124) | **<0.0001** | **<0.0001** | -0.0923 (-0.292-0.109) | **<0.0001** | **<0.0001** |
| 4 | 0.462 (0.196-0.719) | 0.363 (0.0978-0.61) | **<0.0001** | 0.399 (0.128-0.647) | **<0.0001** | **<0.0001** | 0.392 (0.122-0.639) | **<0.0001** | **<0.0001** |
| 5 | 0.488 (0.212-0.702) | -0.0536 (-0.291-0.199) | **<0.0001** | -0.0386 (-0.253-0.196) | 0.065314 | **<0.0001** | -0.0392 (-0.265-0.202) | 0.167311 | **<0.0001** |
| 6 | -0.106 (-0.461-0.201) | -0.218 (-0.593-0.107) | **<0.0001** | -0.191 (-0.578-0.136) | **0.00045** | **<0.0001** | -0.2 (-0.58-0.127) | 0.014856 | **<0.0001** |
| 7 | 0.525 (0.329-0.667) | 0.0332 (-0.235-0.259) | **<0.0001** | 0.217 (0.0334-0.39) | **<0.0001** | **<0.0001** | 0.133 (-0.074-0.34) | **<0.0001** | **<0.0001** |
| 8 | 0.169 (-0.0457-0.364) | -0.0548 (-0.261-0.136) | **<0.0001** | -0.0077 (-0.226-0.221) | **<0.0001** | **<0.0001** | -0.0076 (-0.233-0.199) | **<0.0001** | **<0.0001** |
| 9 | 0.511 (0.226-0.682) | 0.356 (0.0479-0.46) | **<0.0001** | 0.473 (0.16-0.611) | **0.001769** | 0.061 | 0.433 (0.142-0.586) | **0.021672** | **0.012** |
| 10 | 0.0635 (-0.175-0.283) | 0.0417 (-0.178-0.255) | **0.002188531** | 0.0491 (-0.179-0.265) | 0.254996 | 0.053 | 0.0488 (-0.178-0.262) | 0.362472 | **0.031** |
| 11 | 0.208 (-0.0899-0.42) | 0.257 (0.056-0.436) | **0.003441301** | 0.283 (0.0988-0.461) | **0.049167** | **<0.0001** | 0.284 (0.0815-0.451) | 0.137526 | **<0.0001** |
| 12 | 0.0435 (-0.17-0.248) | 0.16 (-0.0904-0.378) | **<0.0001** | 0.129 (-0.109-0.352) | **<0.0001** | **<0.0001** | 0.139 (-0.107-0.362) | **0.001261** | **<0.0001** |
| 13 | 0.902 (0.796-0.943) | 0.801 (0.669-0.878) | **<0.0001** | 0.8 (0.667-0.883) | 0.733962 | **<0.0001** | 0.802 (0.671-0.877) | 0.845486 | **<0.0001** |
| 14 | 0.246 (-0.0636-0.505) | 0.262 (-0.0234-0.493) | **0.042847001** | 0.283 (-0.00315-0.499) | **0.009115** | **<0.0001** | 0.28 (-0.00595-0.503) | **0.009985** | **<0.0001** |
| 15 | 0.319 (0.116-0.512) | 0.223 (0.0311-0.402) | **<0.0001** | 0.244 (0.0496-0.435) | **0.008412** | **<0.0001** | 0.24 (0.0474-0.429) | **0.042516** | **<0.0001** |
| 16 | 0.891 (0.786-0.932) | 0.801 (0.672-0.88) | **<0.0001** | 0.809 (0.719-0.895) | **0.026495** | **<0.0001** | 0.787 (0.697-0.882) | 0.95602 | **<0.0001** |
| 17 | 0.832 (0.708-0.894) | 0.681 (0.505-0.78) | **<0.0001** | 0.72 (0.526-0.821) | **<0.0001** | **<0.0001** | 0.71 (0.521-0.813) | **<0.0001** | **<0.0001** |
| 18 | 0.0108 (-0.25-0.338) | -0.172 (-0.375-0.0601) | **<0.0001** | -0.152 (-0.351-0.0815) | **0.000224** | **<0.0001** | -0.167 (-0.367-0.0668) | 0.22081 | **<0.0001** |
| 19 | 0.273 (0.0802-0.436) | 0.0482 (-0.153-0.208) | **<0.0001** | 0.0544 (-0.15-0.24) | 0.090999 | **<0.0001** | 0.052 (-0.149-0.223) | 0.530315 | **<0.0001** |
| 20 | 0.0169 (-0.237-0.235) | -0.121 (-0.343-0.0949) | **<0.0001** | -0.121 (-0.337-0.116) | 0.47168 | **<0.0001** | -0.118 (-0.346-0.106) | 0.666139 | **<0.0001** |
| 21 | 0.252 (-0.0982-0.531) | -0.0358 (-0.291-0.185) | **<0.0001** | -0.0434 (-0.292-0.176) | 0.434914 | **<0.0001** | -0.0316 (-0.285-0.185) | 0.485224 | **<0.0001** |
| 22 | 0.855 (0.653-0.958) | 0.512 (0.186-0.764) | **<0.0001** | 0.515 (0.218-0.744) | 0.529139 | **<0.0001** | 0.526 (0.224-0.747) | 0.235564 | **<0.0001** |
| 23 | 0.291 (0.107-0.461) | 0.102 (-0.104-0.285) | **<0.0001** | 0.132 (-0.0708-0.32) | **<0.0001** | **<0.0001** | 0.126 (-0.0819-0.31) | **0.003297** | **<0.0001** |
| 24 | 0.251 (-0.0413-0.723) | 0.403 (0.0628-0.746) | 0.283025972 | 0.266 (-0.0359-0.73) | 0.280348 | 0.982 | 0.343 (-0.0154-0.736) | 0.522432 | 0.689 |
| 25 | 0.377 (0.15-0.558) | 0.0711 (-0.127-0.267) | **<0.0001** | 0.11 (-0.108-0.313) | **0.000853** | **<0.0001** | 0.11 (-0.111-0.314) | **0.005182** | **<0.0001** |
| 26 | 0.147 (-0.162-0.462) | -0.0334 (-0.375-0.249) | **<0.0001** | 0.00585 (-0.359-0.289) | 0.079641 | **<0.0001** | -0.0034 (-0.384-0.278) | 0.377979 | **<0.0001** |
| 27 | 0.03 (-0.22-0.291) | 0.035 (-0.234-0.273) | **0.033753272** | 0.0477 (-0.219-0.298) | **0.001446** | 0.321 | 0.046 (-0.221-0.291) | **0.009837** | 0.685 |
| 28 | 0.0294 (-0.277-0.315) | -0.104 (-0.376-0.15) | **<0.0001** | -0.0928 (-0.376-0.192) | **0.006964** | **<0.0001** | -0.0999 (-0.377-0.182) | 0.100271 | **<0.0001** |
| 29 | -0.0366 (-0.241-0.224) | -0.0653 (-0.275-0.162) | **<0.0001** | -0.058 (-0.262-0.165) | 0.367885 | **<0.0001** | -0.0592 (-0.268-0.165) | 0.544921 | **<0.0001** |
| 30 | 0.44 (0.245-0.608) | 0.0461 (-0.212-0.287) | **<0.0001** | 0.164 (-0.106-0.386) | **<0.0001** | **<0.0001** | 0.109 (-0.16-0.343) | **<0.0001** | **<0.0001** |
| 31 | -0.165 (-0.404-0.0832) | -0.136 (-0.385-0.128) | **<0.0001** | -0.117 (-0.373-0.135) | 0.089552 | **<0.0001** | -0.124 (-0.375-0.134) | 0.172639 | **<0.0001** |
| 32 | -0.244 (-0.433-0.0045) | -0.425 (-0.619--0.157) | **<0.0001** | -0.548 (-0.7--0.332) | **<0.0001** | **<0.0001** | -0.528 (-0.682--0.302) | **<0.0001** | **<0.0001** |
| 33 | 0.522 (0.266-0.74) | 0.387 (0.142-0.578) | **<0.0001** | 0.41 (0.196-0.617) | **<0.0001** | **<0.0001** | 0.405 (0.184-0.606) | **0.000587** | **<0.0001** |
| 34 | 0.355 (0.159-0.532) | 0.0375 (-0.185-0.298) | **<0.0001** | 0.117 (-0.121-0.37) | **<0.0001** | **<0.0001** | 0.101 (-0.146-0.351) | **<0.0001** | **<0.0001** |
| 35 | -0.158 (-0.417-0.149) | -0.143 (-0.363-0.15) | **0.046313117** | -0.155 (-0.368-0.132) | 0.824701 | 0.071 | -0.156 (-0.37-0.132) | 0.809933 | 0.072 |
| 36 | 0.0622 (-0.142-0.285) | 0.0293 (-0.199-0.266) | **<0.0001** | 0.0506 (-0.176-0.283) | **0.007884** | **0.01** | 0.043 (-0.183-0.275) | 0.066974 | **0.001** |
| 37 | 0.792 (0.498-0.942) | 0.682 (0.438-0.891) | **<0.0001** | 0.722 (0.435-0.908) | 0.051137 | **<0.0001** | 0.681 (0.426-0.892) | 0.882437 | **<0.0001** |
| 38 | 0.294 (0.032-0.486) | -0.0485 (-0.268-0.204) | **<0.0001** | -0.0925 (-0.297-0.132) | **<0.0001** | **<0.0001** | -0.0793 (-0.287-0.156) | **0.000773** | **<0.0001** |
| 39 | 0.316 (0.111-0.5) | 0.0511 (-0.205-0.271) | **<0.0001** | 0.0592 (-0.193-0.307) | 0.436438 | **<0.0001** | 0.0482 (-0.205-0.283) | 0.589593 | **<0.0001** |
| 40 | 0.0998 (-0.152-0.342) | -0.484 (-0.688--0.219) | **<0.0001** | -0.483 (-0.703--0.218) | 0.513727 | **<0.0001** | -0.492 (-0.705--0.223) | 0.101295 | **<0.0001** |
| 41 | 0.194 (-0.0118-0.379) | -0.124 (-0.33-0.079) | **<0.0001** | -0.118 (-0.331-0.0795) | 0.920019 | **<0.0001** | -0.129 (-0.339-0.0724) | 0.580439 | **<0.0001** |
| 42 | 0.206 (-0.115-0.448) | -0.436 (-0.637--0.166) | **<0.0001** | -0.488 (-0.651--0.221) | **0.011546** | **<0.0001** | -0.472 (-0.651--0.197) | 0.052945 | **<0.0001** |
| 43 | 0.465 (0.244-0.627) | 0.127 (-0.14-0.336) | **<0.0001** | 0.145 (-0.157-0.371) | 0.51108 | **<0.0001** | 0.148 (-0.161-0.365) | 0.560592 | **<0.0001** |
| 44 | 0.282 (0.0779-0.472) | 0.116 (-0.0874-0.324) | **<0.0001** | 0.144 (-0.0651-0.333) | **0.01236** | **<0.0001** | 0.133 (-0.069-0.334) | 0.056366 | **<0.0001** |
| 45 | 0.281 (-0.00258-0.462) | 0.18 (-0.0726-0.365) | **<0.0001** | 0.166 (-0.0708-0.359) | 0.65304 | **<0.0001** | 0.175 (-0.0696-0.364) | 0.797763 | **<0.0001** |
| 46 | 0.443 (0.195-0.624) | -0.0768 (-0.362-0.167) | **<0.0001** | -0.058 (-0.395-0.21) | 0.545517 | **<0.0001** | -0.0659 (-0.401-0.209) | 0.384593 | **<0.0001** |
| 47 | 0.13 (-0.216-0.419) | 0.002 (-0.128-0.187) | 0.079951796 | 0.0428 (-0.112-0.172) | 0.70758 | 0.113 | 0.0382 (-0.16-0.2) | 0.976811 | 0.071 |
| 48 | -0.106 (-0.323-0.123) | -0.0558 (-0.263-0.169) | **<0.0001** | -0.0507 (-0.257-0.169) | 0.321006 | **<0.0001** | -0.0524 (-0.258-0.172) | 0.322377 | **<0.0001** |
| 49 | 0.417 (0.215-0.674) | 0.219 (-0.0648-0.484) | **<0.0001** | 0.204 (-0.0516-0.465) | 0.211558 | **<0.0001** | 0.212 (-0.0554-0.475) | 0.399688 | **<0.0001** |
| 50 | 0.164 (-0.0891-0.375) | 0.118 (-0.129-0.315) | **0.001548773** | 0.149 (-0.081-0.34) | **0.014936** | 0.391 | 0.139 (-0.108-0.338) | 0.162437 | 0.072 |
| 51 | 0.305 (0.0541-0.544) | 0.132 (-0.346-0.442) | **<0.0001** | 0.166 (-0.281-0.476) | **0.01061** | **<0.0001** | 0.164 (-0.304-0.462) | 0.066036 | **<0.0001** |
| 52 | 0.305 (0.0572-0.472) | -0.0366 (-0.248-0.162) | **<0.0001** | -0.0089 (-0.218-0.183) | **0.013813** | **<0.0001** | -0.0105 (-0.236-0.178) | 0.080623 | **<0.0001** |
| 53 | 0.18 (-0.0846-0.44) | 0.606 (0.427-0.764) | **<0.0001** | 0.634 (0.417-0.806) | **0.024699** | **<0.0001** | 0.65 (0.454-0.814) | 0.002779 | **<0.0001** |
| 54 | 0.513 (0.325-0.653) | 0.253 (0.0546-0.428) | **<0.0001** | 0.291 (0.0884-0.47) | **<0.0001** | **<0.0001** | 0.291 (0.0867-0.468) | <0.0001 | **<0.0001** |
| 55 | 0.373 (0.0946-0.582) | 0.0573 (-0.3-0.391) | **<0.0001** | 0.0796 (-0.3-0.41) | 0.375256 | **<0.0001** | 0.0721 (-0.302-0.412) | 0.641011 | **<0.0001** |
| 56 | -0.14 (-0.376-0.105) | -0.428 (-0.61--0.189) | **<0.0001** | -0.451 (-0.638--0.221) | **0.043415** | **<0.0001** | -0.459 (-0.649--0.234) | **0.008042** | **<0.0001** |
| 57 | 0.179 (-0.057-0.417) | -0.257 (-0.496-0.0356) | **<0.0001** | -0.234 (-0.474-0.0538) | **0.027949** | **<0.0001** | -0.243 (-0.487-0.048) | 0.204022 | **<0.0001** |
| 58 | 0.191 (-0.0389-0.382) | 0.0478 (-0.158-0.249) | **<0.0001** | 0.123 (-0.108-0.299) | **<0.0001** | **<0.0001** | 0.0978 (-0.124-0.283) | **0.006575** | **<0.0001** |
| 59 | 0.128 (-0.124-0.355) | 0.213 (-0.049-0.438) | **<0.0001** | 0.23 (-0.0244-0.45) | **0.025923** | **<0.0001** | 0.22 (-0.0358-0.444) | 0.23306 | **<0.0001** |
| 60 | 0.0408 (-0.15-0.264) | -0.11 (-0.3-0.0984) | **<0.0001** | -0.106 (-0.295-0.0967) | 0.739302 | **<0.0001** | -0.109 (-0.301-0.095) | 0.950476 | **<0.0001** |
| 61 | 0.431 (0.202-0.579) | 0.352 (0.159-0.504) | **0.002084749** | 0.336 (0.133-0.514) | 0.889493 | **0.008** | 0.353 (0.125-0.526) | 0.564717 | **0.019** |
| 62 | -0.146 (-0.372-0.0849) | -0.0882 (-0.304-0.126) | **<0.0001** | -0.115 (-0.329-0.101) | **<0.0001** | **<0.0001** | -0.113 (-0.326-0.105) | **<0.0001** | **<0.0001** |
| 63 | 0.311 (0.0805-0.51) | 0.468 (0.253-0.63) | **<0.0001** | 0.442 (0.22-0.606) | 0.068421 | **<0.0001** | 0.46 (0.225-0.62) | 0.362014 | **<0.0001** |
| 64 | 0.145 (-0.101-0.388) | 0.0908 (-0.132-0.306) | **<0.0001** | 0.124 (-0.107-0.338) | **<0.0001** | **<0.0001** | 0.115 (-0.115-0.331) | **<0.0001** | **<0.0001** |
| 65 | 0.109 (-0.123-0.347) | -0.131 (-0.367-0.083) | **<0.0001** | -0.108 (-0.333-0.109) | **0.00736** | **<0.0001** | -0.124 (-0.355-0.103) | 0.21741 | **<0.0001** |
| 66 | 0.476 (0.2-0.715) | 0.18 (-0.0819-0.43) | **<0.0001** | 0.204 (-0.068-0.458) | 0.133177 | **<0.0001** | 0.2 (-0.0674-0.459) | 0.200677 | **<0.0001** |
| 67 | -0.206 (-0.402-0.0074) | -0.291 (-0.504--0.0658) | **<0.0001** | -0.345 (-0.537--0.124) | **<0.0001** | **<0.0001** | -0.333 (-0.536--0.108) | **<0.0001** | **<0.0001** |
| 68 | -0.165 (-0.349-0.0739) | -0.31 (-0.525--0.0859) | **<0.0001** | -0.364 (-0.553--0.14) | **<0.0001** | **<0.0001** | -0.355 (-0.555--0.128) | **<0.0001** | **<0.0001** |
| 69 | 0.228 (-0.0177-0.436) | -0.224 (-0.449--0.0114) | **<0.0001** | -0.225 (-0.414--0.0136) | 0.699279 | **<0.0001** | -0.244 (-0.443--0.0248) | 0.450141 | **<0.0001** |
| 70 | 0.124 (-0.118-0.425) | -0.207 (-0.419-0.112) | **<0.0001** | -0.152 (-0.372-0.16) | **<0.0001** | **<0.0001** | -0.182 (-0.395-0.141) | **0.007219** | **<0.0001** |
| 71 | 0.0457 (-0.165-0.219) | -0.065 (-0.259-0.106) | **<0.0001** | -0.1 (-0.283-0.0635) | **0.000528** | **<0.0001** | -0.0984 (-0.28-0.0739) | **0.002485** | **<0.0001** |
| 72 | 0.206 (-0.0208-0.443) | 0.0597 (-0.136-0.269) | **<0.0001** | 0.0781 (-0.12-0.279) | **0.001477** | **<0.0001** | 0.0782 (-0.127-0.28) | **0.002229** | **<0.0001** |
| 73 | 0.42 (0.195-0.61) | 0.37 (0.122-0.575) | **<0.0001** | 0.41 (0.154-0.598) | **<0.0001** | **<0.0001** | 0.402 (0.148-0.592) | **<0.0001** | **<0.0001** |
| 74 | 0.0425 (-0.216-0.279) | -0.11 (-0.289-0.0916) | **<0.0001** | -0.0992 (-0.281-0.0857) | 0.702501 | **<0.0001** | -0.11 (-0.286-0.0898) | 0.884746 | **<0.0001** |
| 75 | 0.0161 (-0.27-0.287) | -0.118 (-0.365-0.112) | **<0.0001** | -0.111 (-0.337-0.101) | 0.540821 | **<0.0001** | -0.115 (-0.338-0.104) | 0.656924 | **<0.0001** |
| 76 | -0.0102 (-0.188-0.183) | -0.105 (-0.268-0.0735) | **<0.0001** | -0.0764 (-0.232-0.086) | **0.000232** | **<0.0001** | -0.0862 (-0.246-0.0818) | **0.014014** | **<0.0001** |
| 77 | 0.00155 (-0.224-0.25) | -0.097 (-0.295-0.0952) | **<0.0001** | -0.121 (-0.319-0.0952) | **0.000541** | **<0.0001** | -0.125 (-0.328-0.0883) | **<0.0001** | **<0.0001** |
| 78 | 0.0184 (-0.21-0.273) | -0.0523 (-0.257-0.186) | **<0.0001** | -0.0901 (-0.297-0.144) | **<0.0001** | **<0.0001** | -0.0892 (-0.3-0.15) | **<0.0001** | **<0.0001** |
| 79 | -0.159 (-0.439-0.155) | -0.271 (-0.509-0.0253) | **<0.0001** | -0.249 (-0.492-0.0537) | **0.00047** | **<0.0001** | -0.258 (-0.503-0.0483) | **0.048042** | **<0.0001** |
| 80 | -0.112 (-0.306-0.0894) | -0.18 (-0.382-0.0355) | **<0.0001** | -0.166 (-0.364-0.0421) | **0.00064** | **<0.0001** | -0.17 (-0.369-0.0388) | **0.032351** | **<0.0001** |
| 81 | 0.124 (-0.113-0.375) | 0.134 (-0.0985-0.342) | 0.386166324 | 0.126 (-0.106-0.342) | 0.570828 | 0.162 | 0.125 (-0.105-0.343) | 0.461171 | 0.117 |
| 82 | 0.0832 (-0.166-0.317) | 0.0113 (-0.232-0.242) | **<0.0001** | 0.0392 (-0.198-0.257) | **0.000326** | **<0.0001** | 0.0347 (-0.208-0.256) | **0.007599** | **<0.0001** |
| 83 | 0.249 (0.0073-0.507) | -0.11 (-0.378-0.149) | **<0.0001** | -0.0715 (-0.363-0.169) | 0.209581 | **<0.0001** | -0.0938 (-0.381-0.158) | 0.647611 | **<0.0001** |
| 84 | 0.0511 (-0.172-0.273) | -0.128 (-0.402-0.094) | **<0.0001** | -0.109 (-0.37-0.108) | **0.002418** | **<0.0001** | -0.114 (-0.385-0.101) | 0.075758 | **<0.0001** |
| 85 | 0.317 (0.0741-0.504) | -0.0739 (-0.35-0.155) | **<0.0001** | -0.0629 (-0.349-0.154) | 0.817348 | **<0.0001** | -0.0582 (-0.404-0.148) | 0.893043 | **<0.0001** |
| 86 | 0.367 (0.141-0.55) | 0.0982 (-0.122-0.292) | **<0.0001** | 0.117 (-0.106-0.32) | **0.010052** | **<0.0001** | 0.112 (-0.109-0.316) | **0.027298** | **<0.0001** |
| 87 | 0.1 (-0.1-0.323) | 0.21 (0.00825-0.394) | **<0.0001** | 0.191 (-0.0126-0.366) | **0.00047** | **<0.0001** | 0.201 (-0.00165-0.381) | 0.099788 | **<0.0001** |
| 88 | 0.112 (-0.187-0.38) | -0.236 (-0.509-0.0753) | **<0.0001** | -0.182 (-0.46-0.152) | **<0.0001** | **<0.0001** | -0.205 (-0.478-0.134) | **<0.0001** | **<0.0001** |
| 89 | -0.0434 (-0.265-0.173) | -0.132 (-0.281-0.0901) | **<0.0001** | -0.106 (-0.281-0.0881) | 0.454808 | **<0.0001** | -0.0981 (-0.284-0.0951) | 0.460999 | **<0.0001** |
| 90 | 0.0594 (-0.194-0.278) | -0.177 (-0.413-0.065) | **<0.0001** | -0.164 (-0.393-0.0952) | 0.261732 | **<0.0001** | -0.176 (-0.403-0.0853) | 0.619745 | **<0.0001** |
| 91 | 0.202 (-0.0597-0.487) | 0.26 (-0.0112-0.527) | **<0.0001** | 0.267 (-0.002-0.523) | 0.256223 | **<0.0001** | 0.266 (-0.00115-0.529) | 0.25681 | **<0.0001** |
| 92 | 0.816 (0.63-0.906) | 0.531 (0.32-0.681) | **<0.0001** | 0.602 (0.387-0.751) | **<0.0001** | **<0.0001** | 0.573 (0.364-0.729) | **<0.0001** | **<0.0001** |
| 93 | 0.529 (0.235-0.725) | 0.309 (0.0022-0.552) | **<0.0001** | 0.348 (0.0091-0.594) | **<0.0001** | **<0.0001** | 0.335 (0.01-0.579) | **0.001013** | **<0.0001** |
| 94 | 0.082 (-0.196-0.346) | -0.00805 (-0.225-0.226) | **<0.0001** | 0.0159 (-0.222-0.247) | 0.34341 | **<0.0001** | 0.02 (-0.223-0.258) | 0.154436 | **<0.0001** |
| 95 | 0.59 (0.322-0.769) | 0.472 (0.23-0.656) | **<0.0001** | 0.511 (0.247-0.694) | **<0.0001** | **<0.0001** | 0.506 (0.245-0.685) | **<0.0001** | **<0.0001** |
| 96 | -0.0055 (-0.23-0.203) | -0.117 (-0.334-0.085) | **<0.0001** | -0.101 (-0.307-0.0844) | 0.025495 | **<0.0001** | -0.106 (-0.319-0.0867) | 0.099657 | **<0.0001** |
| 97 | 0.156 (-0.106-0.425) | 0.0039 (-0.214-0.192) | **<0.0001** | 0.0334 (-0.186-0.202) | 0.120736 | **<0.0001** | 0.011 (-0.19-0.186) | 0.504343 | **<0.0001** |
| 98 | -0.0141 (-0.282-0.247) | 0.123 (-0.168-0.39) | **<0.0001** | 0.131 (-0.172-0.406) | 0.843775 | **<0.0001** | 0.0952 (-0.166-0.404) | 0.883307 | **<0.0001** |
| 99 | -0.0422 (-0.287-0.321) | -0.344 (-0.532--0.104) | **<0.0001** | -0.398 (-0.58--0.117) | **<0.0001** | **<0.0001** | -0.392 (-0.576--0.128) | **<0.0001** | **<0.0001** |
| 100 | 0.19 (-0.0727-0.401) | 0.153 (-0.038-0.348) | 0.174119844 | 0.172 (-0.0318-0.375) | 0.101684 | 0.869 | 0.165 (-0.0493-0.376) | 0.354481 | 0.659 |

*Highlighted p values less than 0.05. AMP, pulse amplitude; IQR, interquartile range; PAx, pulse amplitude index;*

## Table C3. Windowing Differences on CPPopt

| **Patient** | **PRx – CPPopt** | **PAx Rectangular – CPPopt** | | **PAx Hanning – CPPopt** | | | **PAx Chebyshev – CPPopt** | | |
| --- | --- | --- | --- | --- | --- | --- | --- | --- | --- |
|  | **Median (IQR)** | **Median (IQR)** | **PRx and PAx P value** | **Median (IQR)** | **PAx Rectangular and Hanning p value** | **PRx and PAx Hanning p value** | **Median (IQR)** | **PAx Rectangular and Chebyshev p value** | **PRx and PAx Hanning p value** |
| 1 | 79.4 (74.3-84.2) | 76.1 (71.4-81) | **<0.0001** | 75.5 (70.8-80.4) | **<0.0001** | **<0.0001** | 75.4 (71.1-80.6) | **0.005** | **<0.0001** |
| 2 | 67 (64.4-70.8) | 71.4 (64.8-75.9) | **<0.0001** | 70.7 (68.6-77.5) | **0.003** | **<0.0001** | 71.4 (68.2-75.8) | **0.056** | **<0.0001** |
| 3 | 78.5 (71.6-82.8) | 83.6 (78.6-89.9) | **<0.0001** | 86.7 (81.4-92.5) | **<0.0001** | **<0.0001** | 86 (81.3-91.6) | **<0.0001** | **<0.0001** |
| 4 | 84.8 (75.3-90.6) | 82.5 (74-88.4) | **<0.0001** | 81.4 (74.9-88.7) | 0.14 | **<0.0001** | 81.6 (75.3-88.5) | 0.139 | **<0.0001** |
| 5 | 60.8 (58.2-63.6) | 64.5 (60.8-69.4) | **<0.0001** | 65.8 (60.6-70.1) | 0.097 | **<0.0001** | 65.7 (58.9-69.8) | 0.271 | **<0.0001** |
| 6 | 102 (94.8-109) | 103 (97.2-108) | **<0.0001** | 103 (96.6-107) | **0.031** | 0.147 | 102 (96.4-108) | **<0.0001** | 0.941 |
| 7 | 76.6 (75.2-101) | 79.5 (76.2-86.4) | 0.191 | 83.6 (78.4-90.5) | **<0.0001** | **<0.0001** | 79.7 (77.2-88.6) | **<0.0001** | **0.001** |
| 8 | 85.9 (84.8-101) | 95.2 (83.8-101) | 0.561 | 90.4 (86-100) | **0.043** | **<0.0001** | 90.7 (84.2-99.3) | **0.002** | 0.053 |
| 9 | 72.4 (69.7-81.7) | 72.8 (69-78.4) | **<0.0001** | 73.1 (69.1-82.8) | **0.004** | 0.073 | 73 (69.1-82.8) | **0.01** | **0.012** |
| 10 | 84.8 (84.6-85.5) | 88.7 (88.5-89.7) | **<0.0001** | 87.9 (87.9-88.2) | **<0.0001** | **<0.0001** | 88.5 (88.5-88.8) | **<0.0001** | **<0.0001** |
| 11 | 77 (73.9-79.9) | 79.4 (75-83.4) | **<0.0001** | 78.8 (73.8-82.7) | **<0.0001** | **<0.0001** | 78.4 (73.4-82.6) | **<0.0001** | **<0.0001** |
| 12 | 90.6 (89.8-91.2) | 85.2 (84.5-88.5) | **<0.0001** | 82.6 (82.2-83.4) | **<0.0001** | **<0.0001** | 82.4 (82.2-83.2) | **<0.0001** | **<0.0001** |
| 13 | 79.3 (73.5-83.1) | 77.2 (70.2-81.9) | **<0.0001** | 76.2 (70.4-81.7) | **<0.0001** | **<0.0001** | 76.1 (70.5-81.8) | **<0.0001** | **<0.0001** |
| 14 | 73.7 (64.8-74.5) | 67.6 (65.7-68.4) | **0.004** | 68.7 (66.2-70.4) | **<0.0001** | **0.011** | 68.5 (65.7-69.5) | **0.001** | **0.007** |
| 15 | 70 (60.7-70.1) | 66.6 (52.2-66.8) | 0.082 | 68.1 (50.5-68.8) | 0.442 | 0.082 | 67.9 (51.6-68.6) | 0.442 | 0.082 |
| 16 | 72 (67.4-88.8) | 73.9 (70.4-91.6) | **<0.0001** | 74.2 (71.9-89.9) | 0.127 | **<0.0001** | 75.4 (71.1-89.5) | 0.051 | **<0.0001** |
| 17 | 83.9 (83.6-84.5) | 70.9 (70-86.2) | **0.001** | 84.2 (83.3-85.3) | **<0.0001** | 0.66 | 84.3 (83.6-87.4) | **<0.0001** | 0.233 |
| 18 | 75.1 (73.1-79.4) | 75.7 (69.6-77.2) | 0.482 | 76.2 (72-77.8) | **0.023** | 0.594 | 75.9 (71-78) | **0.008** | 0.972 |
| 19 | 73.9 (69.1-80.3) | 84.9 (81-92) | **<0.0001** | 85 (79.5-88.6) | **<0.0001** | **<0.0001** | 85.2 (80.4-89.3) | **<0.0001** | **<0.0001** |
| 20 | 61.7 (60.6-63) | 61.8 (60.4-67) | 0.209 | 61.1 (59.4-67.4) | **<0.0001** | **0.001** | 59.8 (58.6-67.4) | **<0.0001** | **<0.0001** |
| 21 | 75 (72.8-78.4) | 80.5 (77.8-82.5) | **<0.0001** | 80.3 (75.2-81.8) | 0.428 | **<0.0001** | 80.2 (75.6-81.7) | 0.308 | **<0.0001** |
| 22 | 73.5 (69.9-84.9) | 84.6 (74.3-88.4) | **<0.0001** | 80.7 (77.8-88.6) | 0.056 | **<0.0001** | 81 (78.1-88.7) | **0.017** | **<0.0001** |
| 23 | 86.1 (81.9-93.6) | 83.5 (81.1-85.1) | **<0.0001** | 83.2 (81-84.2) | **0.004** | **<0.0001** | 83.5 (82.4-84.6) | 0.616 | **<0.0001** |
| 24 | 70.8 (65-76.7) | 68.8 (62.9-75.8) | **0.022** | 70.1 (60.4-78.1) | 0.655 | **0.027** | 69.5 (61.3-77) | 0.865 | **0.019** |
| 25 | 67.6 (64.9-70.4) | 69.8 (61.9-79.2) | **<0.0001** | 67.4 (63.8-80.1) | **0.025** | **<0.0001** | 67.4 (62.7-79.4) | 0.706 | **0.048** |
| 26 | 75.7 (69.8-78) | 73.2 (69-76) | **<0.0001** | 73.3 (69.6-76) | 0.251 | **<0.0001** | 72.8 (69.6-76.2) | 0.669 | **<0.0001** |
| 27 | 75 (72.3-77.7) | 85.4 (82.5-87.4) | **<0.0001** | 87 (84.5-90.7) | **<0.0001** | **<0.0001** | 86.4 (84.2-89.6) | **<0.0001** | **<0.0001** |
| 28 | 67.7 (62.3-69.6) | 74.4 (66.8-78.4) | **<0.0001** | 71 (69.7-77.3) | **0.046** | **<0.0001** | 71.8 (69-77.2) | 0.027 | **<0.0001** |
| 29 | 73.3 (67-77.9) | 67.8 (62.6-74.2) | **<0.0001** | 68.5 (63.3-73.8) | 0.106 | **<0.0001** | 68.2 (64.1-71.9) | 0.307 | **<0.0001** |
| 30 | 69.3 (68.6-72) | 76.4 (70-81.5) | **<0.0001** | 75.3 (71.3-78.9) | 0.105 | **<0.0001** | 75.3 (70.5-79.1) | **0.03** | **<0.0001** |
| 31 | 82.9 (77.4-85.3) | 80.1 (76.3-83) | **<0.0001** | 78.5 (77.4-81.7) | 0.181 | **<0.0001** | 77.6 (76-81) | **<0.0001** | **<0.0001** |
| 32 | 66.5 (61.4-69.7) | 63.6 (61.3-65.2) | **0.039** | 68.4 (66.5-77.6) | **<0.0001** | **<0.0001** | 67.4 (65.6-70.7) | **<0.0001** | **<0.0001** |
| 33 | 69.1 (61-75.9) | 71 (66.9-75.3) | **<0.0001** | 68.3 (66.9-75.6) | 0.532 | **<0.0001** | 71.8 (66.4-75.6) | 0.422 | **<0.0001** |
| 34 | 74.9 (58-77.4) | 65.4 (55.5-74.8) | **<0.0001** | 67.3 (56.5-74.4) | **0.015** | **<0.0001** | 66.3 (56.2-74.8) | **0.021** | **<0.0001** |
| 35 | 89.7 (85.2-95.5) | 91.1 (80.8-99.7) | **<0.0001** | 90.6 (79.9-98.4) | **0.002** | 0.468 | 90.1 (79.4-99.2) | **0.015** | 0.056 |
| 36 | 64.6 (64-69.2) | 70.7 (69.1-72.9) | **0.021** | 66.9 (65.5-68.5) | **0.001** | 0.848 | 72.7 (72.2-72.9) | 0.259 | **0.002** |
| 37 | 65.3 (64.2-67.1) | 74.2 (72.4-81.1) | **<0.0001** | 76.8 (74.3-80.2) | **<0.0001** | **<0.0001** | 76 (73.5-79.9) | **<0.0001** | **<0.0001** |
| 38 | 91.3 (90.7-91.3) | 88.6 (84.2-90.5) | **<0.0001** | 82.6 (80.3-84.3) | **<0.0001** | **<0.0001** | 81.3 (78.9-82.7) | **<0.0001** | **<0.0001** |
| 39 | 78.4 (75.9-101) | 83.8 (77.8-106) | **0.001** | 84.4 (73.1-102) | 0.061 | 0.218 | 83.2 (73.3-103) | **0.026** | **0.083** |
| 40 | 76.6 (72.6-85) | 76.7 (65.7-77.2) | **0.005** | 77 (70.1-77.3) | 0.28 | **0.017** | 77.3 (68.8-77.6) | **0.001** | **0.018** |
| 41 | 81.7 (78.5-93.6) | 83.5 (77.1-97.1) | 0.806 | 83.2 (77.1-94.8) | **0.008** | 0.117 | 83.4 (77-94.4) | **0.072** | 0.371 |
| 42 | 75.4 (74.8-76) | 71.3 (70.4-73.7) | **<0.0001** | 74.2 (73.9-74.3) | **<0.0001** | **<0.0001** | 74.2 (72.1-75.4) | **<0.0001** | **<0.0001** |
| 43 | 70.8 (66.2-74.2) | 74.4 (72.4-78.3) | **<0.0001** | 75.8 (72-79.4) | **0.004** | **<0.0001** | 74.7 (71.2-77.7) | 0.17 | **<0.0001** |
| 44 | 86.9 (81.7-91.9) | 81.7 (76.3-86.5) | **<0.0001** | 81.7 (74.3-86.9) | **<0.0001** | **<0.0001** | 81.8 (74.4-86.7) | **0.005** | **<0.0001** |
| 45 | 75.7 (74.9-77.1) | 76.3 (74.1-82) | **<0.0001** | 77.4 (75.4-82.1) | **<0.0001** | **<0.0001** | 76.9 (74.1-82.1) | 0.118 | **<0.0001** |
| 46 | 115 (115-115) | 108 (108-109) | **<0.0001** | 113 (113-114) | **<0.0001** | **<0.0001** | 112 (112-113) | **<0.0001** | **<0.0001** |
| 47 | 79.6 (78.4-82.2) | 81.1 (78.6-85.6) | **<0.0001** | 83.2 (78.6-86.2) | **0.002** | **<0.0001** | 83 (78.7-86.2) | **<0.0001** | **<0.0001** |
| 48 | 63.6 (63.4-63.7) | 67.7 (67.6-68) | **<0.0001** | 66.7 (66.7-66.7) | **<0.0001** | **<0.0001** | 66.5 (66.5-66.6) | **<0.0001** | **<0.0001** |
| 49 | 78.4 (75.4-80.7) | 84.4 (83-86) | **<0.0001** | 83 (82-83.4) | **<0.0001** | **<0.0001** | 83.2 (82.3-83.8) | **<0.0001** | **<0.0001** |
| 50 | 90.9 (86.6-91) | 81.9 (77.4-84.1) | **<0.0001** | 82.7 (80-83) | **<0.0001** | **<0.0001** | 82.3 (79.5-83.1) | **0.002** | **<0.0001** |
| 51 | 69.1 (67.1-71.2) | 80.2 (75.7-80.8) | **<0.0001** | 81 (78.6-81.9) | **<0.0001** | **<0.0001** | 80.6 (77.7-80.9) | **<0.0001** | **<0.0001** |
| 52 | 72.5 (71.8-75.4) | 71.7 (70.2-74.8) | **0.007** | 74.8 (70.1-75.9) | **<0.0001** | 0.574 | 74.6 (70.3-76.2) | **<0.0001** | 0.134 |
| 53 | 63.8 (63-65.1) | 68.7 (67.3-72.2) | **<0.0001** | 70.3 (67-74.7) | **<0.0001** | **<0.0001** | 69.3 (67-73) | 0.145 | **<0.0001** |
| 54 | 85.1 (81.1-86.9) | 87.3 (76.5-88.1) | 0.357 | 87.2 (77.3-90.4) | **0.002** | 0.063 | 87.4 (76.9-90.4) | **0.003** | 0.078 |
| 55 | 68.7 (63.4-72.7) | 70.8 (67.4-73) | **<0.0001** | 72.5 (66.3-74.2) | **<0.0001** | **<0.0001** | 72 (66.6-73.6) | **<0.0001** | **<0.0001** |
| 56 | 86.3 (83.6-88.1) | 87 (84.7-89.2) | **<0.0001** | 86.6 (84.5-89.7) | 0.707 | **<0.0001** | 86.8 (84.1-89.2) | 0.482 | **<0.0001** |
| 57 | 82.7 (82.4-82.7) | 79.2 (78.7-81.6) | **<0.0001** | 73.3 (72.9-73.6) | **<0.0001** | **<0.0001** | 74.1 (73.9-74.3) | **<0.0001** | **<0.0001** |
| 58 | 74.8 (67-80.8) | 78.9 (68.8-87.2) | **<0.0001** | 78.5 (68-86.8) | 0.416 | **<0.0001** | 79.1 (69.1-87) | 0.949 | **<0.0001** |
| 59 | 80.3 (67.6-81) | 68.5 (62.5-68.9) | **<0.0001** | 67.4 (63-67.8) | **0.01** | **<0.0001** | 67.7 (62.9-68) | **0.007** | **<0.0001** |
| 60 | 100 (95-102) | 98.9 (95.9-102) | 0.513 | 98.7 (95.8-103) | 0.297 | **0.034** | 98.9 (96.5-102) | 0.145 | 0.171 |
| 61 | 69.3 (67-71.2) | 69.3 (66.8-71.6) | 0.13 | 68.1 (65.7-70.5) | **<0.0001** | **<0.0001** | 68.2 (66.2-70.8) | **<0.0001** | **<0.0001** |
| 62 | 84.3 (73.9-105) | 79.7 (70.9-92.9) | **<0.0001** | 74.9 (67.3-99.1) | 0.349 | **<0.0001** | 75.3 (68.2-96.3) | 0.878 | **<0.0001** |
| 63 | 71 (67.2-75.3) | 78.6 (69.2-83.6) | **<0.0001** | 78.4 (69.2-84.2) | 0.215 | **<0.0001** | 78.6 (68.7-83.2) | 0.051 | **<0.0001** |
| 64 | 70.9 (66.6-73.2) | 79.8 (78.6-81.4) | **<0.0001** | 78.7 (75.5-81) | **<0.0001** | **<0.0001** | 77.9 (76-80.9) | **<0.0001** | **<0.0001** |
| 65 | 93.5 (93.4-93.5) | 102 (100-102) | **<0.0001** | 101 (95.8-101) | **<0.0001** | **<0.0001** | 101 (96.6-101) | **<0.0001** | **<0.0001** |
| 66 | 75.8 (72.3-77.7) | 86.4 (83.5-88.2) | **<0.0001** | 86.3 (80.7-89.3) | 0.273 | **<0.0001** | 86.1 (80.6-88.9) | 0.077 | **<0.0001** |
| 67 | 67.7 (65.4-68.9) | 71.2 (69.1-71.9) | **<0.0001** | 71.2 (67.5-72.2) | 0.726 | **<0.0001** | 71.3 (67.5-72.2) | 0.521 | **<0.0001** |
| 68 | 81.4 (65.3-86.8) | 82 (76.5-85.9) | **<0.0001** | 80.4 (75.9-86.4) | **<0.0001** | **<0.0001** | 80.4 (74.7-86) | **<0.0001** | **<0.0001** |
| 69 | 77.3 (74.6-80.2) | 75.8 (73.1-82.4) | **0.002** | 75.4 (71.4-81.6) | **<0.0001** | **<0.0001** | 75.1 (72.4-81.9) | **0.001** | **<0.0001** |
| 70 | 63 (61.6-66.4) | 66.5 (62.2-69.9) | **<0.0001** | 67.2 (63.9-73.6) | **<0.0001** | **<0.0001** | 66.6 (61.5-68.3) | **0.001** | **<0.0001** |
| 71 | 83.3 (79.1-85.3) | 82.4 (80.8-83.4) | **<0.0001** | 82.7 (80.4-83.7) | **0.002** | **<0.0001** | 82.3 (79.6-83.5) | 0.872 | **<0.0001** |
| 72 | 67.2 (64.7-72) | 66.1 (64.2-78.3) | **0.005** | 65.1 (64.2-77.7) | **0.044** | **0.05** | 65 (63.3-77.6) | **<0.0001** | 0.971 |
| 73 | 73.9 (68.6-76.9) | 77.6 (74.7-79.6) | **<0.0001** | 77.6 (74.6-81.2) | 0.268 | **<0.0001** | 77.9 (74.9-81.4) | **0.02** | **<0.0001** |
| 74 | 73.5 (72.3-77.2) | 76.2 (71.2-79) | **<0.0001** | 75.4 (69.8-79.5) | 0.073 | **0.008** | 76.4 (69.8-79.6) | 0.951 | **<0.0001** |
| 75 | 69 (65.4-75.3) | 69.8 (67-76.5) | **<0.0001** | 69.8 (67.6-74.4) | 0.403 | **<0.0001** | 69.7 (67.3-76.2) | 0.972 | **<0.0001** |
| 76 | 65.9 (61.8-71.4) | 71 (65.9-75.5) | **<0.0001** | 70.3 (64.5-73.8) | **<0.0001** | **<0.0001** | 70.2 (64.7-74.2) | **<0.0001** | **<0.0001** |
| 77 | 70.5 (68-73.6) | 73.2 (66.5-78.3) | **<0.0001** | 71 (67.7-77.4) | 0.77 | **<0.0001** | 72.4 (67-77.4) | 0.173 | **<0.0001** |
| 78 | 70.8 (66.3-72.9) | 74.3 (72.4-78.7) | **<0.0001** | 73.5 (68.2-78.8) | **<0.0001** | **<0.0001** | 74.2 (68.8-79) | 0.054 | **<0.0001** |
| 79 | 67.3 (66.6-67.4) | 91.5 (87.6-91.5) | **0.011** | 92 (88.5-92) | 0.196 | **0.011** | 92 (88.7-92) | 0.196 | **0.011** |
| 80 | 71.3 (69.4-74.5) | 74.8 (72-78.5) | **<0.0001** | 74.3 (72.1-81.7) | 0.991 | **<0.0001** | 74.8 (71.6-81.5) | 0.633 | **<0.0001** |
| 81 | 67.2 (64.8-80.2) | 69.1 (66.2-78) | **0.022** | 69.2 (64.3-77.6) | **<0.0001** | **0.003** | 69.2 (65.1-76.7) | **0.001** | 0.089 |
| 82 | 68.4 (65.5-75) | 70.8 (67.5-77) | **<0.0001** | 72 (69-77) | **<0.0001** | **<0.0001** | 71.9 (68.6-76.9) | **<0.0001** | **<0.0001** |
| 83 | 78.5 (75.7-82.4) | 82.4 (80.1-85.3) | **<0.0001** | 83.4 (80.4-86.2) | **<0.0001** | **<0.0001** | 82.5 (80-85.5) | **0.009** | **<0.0001** |
| 84 | 69.4 (66.2-80.8) | 86.2 (85.2-88.6) | **<0.0001** | 73.5 (73.4-73.6) | **<0.0001** | **<0.0001** | 73.3 (73.1-73.3) | **<0.0001** | **<0.0001** |
| 85 | 66.4 (63.1-69) | 82.8 (64.8-87.8) | **<0.0001** | 84.6 (74-88) | **0.006** | **<0.0001** | 84.5 (70.7-88.2) | **0.014** | **<0.0001** |
| 86 | 74.7 (69.5-81) | 71.2 (66.8-79.7) | **<0.0001** | 71.6 (65.4-78.1) | **0.013** | **<0.0001** | 71.2 (65.9-79.5) | 0.113 | **<0.0001** |
| 87 | 71.7 (67.5-71.9) | 67.7 (66.5-69.4) | **<0.0001** | 68.9 (66.6-69.6) | **<0.0001** | **<0.0001** | 69.2 (67-69.7) | **<0.0001** | **<0.0001** |
| 88 | 89.4 (80-90.1) | 92.2 (81.3-94.1) | **<0.0001** | 91.1 (80.1-94.4) | **0.021** | **<0.0001** | 91.4 (80.5-94.4) | **<0.0001** | **<0.0001** |
| 89 | 90 (88.2-92.6) | 89.4 (88.9-91.1) | 0.069 | 92.5 (89.5-93.4) | **<0.0001** | **<0.0001** | 93.4 (92.6-94) | **<0.0001** | **<0.0001** |
| 90 | 77.4 (72.5-85) | 68.8 (65.6-80.4) | **<0.0001** | 69.4 (65.7-80.4) | 0.688 | **<0.0001** | 69.8 (65.4-80.6) | 0.704 | **<0.0001** |
| 91 | 65.3 (64.7-66.1) | 69.6 (63.3-72.9) | **<0.0001** | 68.6 (62.2-73.2) | 0.519 | **<0.0001** | 70.4 (62.6-74.4) | 0.249 | **<0.0001** |
| 92 | 71.2 (69.7-71.9) | 72 (71.2-73.5) | **<0.0001** | 71.7 (71.1-73.4) | 0.108 | **<0.0001** | 71.4 (70.4-72.1) | **<0.0001** | **0.031** |
| 93 | 64.4 (62.8-70) | 72.3 (68.7-74.9) | **<0.0001** | 73.7 (70.3-81.9) | **<0.0001** | **<0.0001** | 72.6 (69.9-79.3) | **<0.0001** | **<0.0001** |
| 94 | 66.7 (65.7-67.9) | 68 (67.2-69.5) | **<0.0001** | 67.4 (66.9-69.5) | **0.02** | **<0.0001** | 68.3 (66.6-68.9) | **<0.0001** | **<0.0001** |

*Note in some patients CPPopt could not be found as thus these patients were removed. Highlighted p values less than 0.05. AMP, pulse amplitude; CPPopt, optimal cerebral perfusion pressure; IQR, interquartile range; PAx, pulse amplitude index;*

# References

1. embs11_lori. Highlights in the History of the Fourier Transform. IEEE Pulse. Published January 25, 2016. Accessed September 9, 2022. https://www.embs.org/pulse/articles/highlights-in-the-history-of-the-fourier-transform/

2. Mintchev M. Sampling Theorem and Aliasing in Biomedical Signal Processing. In: *Wiley Encyclopedia of Biomedical Engineering*. John Wiley & Sons, Ltd; 2006. doi:10.1002/9780471740360.ebs0036

3. Froese L, Dian J, Batson C, Gomez A, Unger B, Zeiler FA. The impact of hypertonic saline on cerebrovascular reactivity and compensatory reserve in traumatic brain injury: an exploratory analysis. *Acta Neurochir (Wien)*. Published online September 21, 2020:1-11. doi:10.1007/s00701-020-04579-0

4. Froese L, Dian J, Batson C, et al. The Impact of Vasopressor and Sedative Agents on Cerebrovascular Reactivity and Compensatory Reserve in Traumatic Brain Injury: An Exploratory Analysis. *Neurotrauma Reports*. 2020;1(1):157-168. doi:10.1089/neur.2020.0028

5. Froese L, Gomez A, Sainbhi AS, et al. Continuous Determination of the Optimal Bispectral Index Value Based on Cerebrovascular Reactivity in Moderate/Severe Traumatic Brain Injury: A Retrospective Observational Cohort Study of a Novel Individualized Sedation Target. *Crit Care Explor*. 2022;4(3):e0656. doi:10.1097/CCE.0000000000000656

6. Batson C, Gomez A, Sainbhi AS, Froese L, Zeiler FA. Association of Age and Sex With Multi-Modal Cerebral Physiology in Adult Moderate/Severe Traumatic Brain Injury: A Narrative Overview and Future Avenues for Personalized Approaches. [Review]. *Frontiers in Pharmacology*. 2021;1:676154. doi:10.3389/fphar.2021.676154

7. Sorrentino E, Diedler J, Kasprowicz M, et al. Critical Thresholds for Cerebrovascular Reactivity After Traumatic Brain Injury. *Neurocrit Care*. 2012;16(2):258-266. doi:10.1007/s12028-011-9630-8

8. Zeiler FA, Donnelly J, Smielewski P, Menon DK, Hutchinson PJ, Czosnyka M. Critical Thresholds of Intracranial Pressure-Derived Continuous Cerebrovascular Reactivity Indices for Outcome Prediction in Noncraniectomized Patients with Traumatic Brain Injury. *Journal of Neurotrauma*. 2018;35(10):1107-1115. doi:10.1089/neu.2017.5472

9. Zeiler FA, Ercole A, Cabeleira M, et al. Comparison of Performance of Different Optimal Cerebral Perfusion Pressure Parameters for Outcome Prediction in Adult Traumatic Brain Injury: A Collaborative European NeuroTrauma Effectiveness Research in Traumatic Brain Injury (CENTER-TBI) Study. *J Neurotrauma*. 2019;36(10):1505-1517. doi:10.1089/neu.2018.6182

10. Zeiler FA, Donnelly J, Calviello L, Smielewski P, Menon DK, Czosnyka M. Pressure Autoregulation Measurement Techniques in Adult Traumatic Brain Injury, Part II: A Scoping Review of Continuous Methods. *Journal of Neurotrauma*. 2017;34(23):3224-3237. doi:10.1089/neu.2017.5086

11. Aries MJH, Czosnyka M, Budohoski KP, et al. Continuous monitoring of cerebrovascular reactivity using pulse waveform of intracranial pressure. *Neurocrit Care*. 2012;17(1):67-76. doi:10.1007/s12028-012-9687-z

12. Sorrentino E, Diedler J, Kasprowicz M, et al. Critical Thresholds for Cerebrovascular Reactivity After Traumatic Brain Injury. *Neurocrit Care*. 2012;16(2):258-266. doi:10.1007/s12028-011-9630-8

13. Budohoski KP, Czosnyka M, de Riva N, et al. The Relationship Between Cerebral Blood Flow Autoregulation and Cerebrovascular Pressure Reactivity After Traumatic Brain Injury: *Neurosurgery*. 2012;71(3):652-661. doi:10.1227/NEU.0b013e318260feb1

14. Czosnyka M, Czosnyka Z, Smielewski P. Pressure reactivity index: journey through the past 20 years. *Acta Neurochir*. 2017;159(11):2063-2065. doi:10.1007/s00701-017-3310-1

15. Liu X, Maurits NM, Aries MJH, et al. Monitoring of Optimal Cerebral Perfusion Pressure in Traumatic Brain Injured Patients Using a Multi-Window Weighting Algorithm. *J Neurotrauma*. 2017;34(22):3081-3088. doi:10.1089/neu.2017.5003

16. Depreitere B, Güiza F, Van den Berghe G, et al. Pressure autoregulation monitoring and cerebral perfusion pressure target recommendation in patients with severe traumatic brain injury based on minute-by-minute monitoring data. *J Neurosurg*. 2014;120(6):1451-1457. doi:10.3171/2014.3.JNS131500

17. Donnelly J, Czosnyka M, Adams H, et al. Individualizing Thresholds of Cerebral Perfusion Pressure Using Estimated Limits of Autoregulation. *Crit Care Med*. 2017;45(9):1464-1471. doi:10.1097/CCM.0000000000002575
